# Immune cell atlas of cholangiocarcinomas reveals distinct tumor microenvironments and associated prognoses

**DOI:** 10.1186/s13045-022-01253-z

**Published:** 2022-03-28

**Authors:** Tao Xia, Keyu Li, Nan Niu, Yingkuan Shao, Ding Ding, Dwayne L. Thomas, Hao Jing, Kenji Fujiwara, Haijie Hu, Arsen Osipov, Chunhui Yuan, Christopher L. Wolfgang, Elizabeth D. Thompson, Robert A. Anders, Jin He, Yiping Mou, Adrian G. Murphy, Lei Zheng

**Affiliations:** 1 Department of Oncology, Johns Hopkins University School of Medicine, 1650 Orleans Street, CRB1 Room 351, Baltimore, MD 21231 USA; 2 Department of Surgery, Johns Hopkins University School of Medicine, Baltimore, MD USA; 3 The Sidney Kimmel Cancer Center, Johns Hopkins University School of Medicine, Baltimore, MD USA; 4 The Bloomberg-Kimmel Institute for Cancer Immunotherapy, Johns Hopkins University School of Medicine, Baltimore, MD USA; 5 The Pancreatic Cancer Precision Medicine Center of Excellence Program, Johns Hopkins University School of Medicine, Baltimore, MD USA; 6 Department of Pathology, Johns Hopkins University School of Medicine, Baltimore, MD USA; 7 Present Address: Department of Gastrointestinal and Pancreatic Surgery, Department of General Surgery, and Cancer Center, The Zhejiang Provincial People’s Hospital and the Affiliated People’s Hospital of Hangzhou Medical College, Hangzhou, China; 8 Present Address: Department of Pancreatic Surgery, West China Hospital, Sichuan University, Chengdu, China; 9 Present Address: The Zhejiang University Second Affiliated Hospital, Hangzhou, China

**Keywords:** Cholangiocarcinoma, Tumor microenvironment, PD-1, PD-L1, Effector T cells, T regulator cells, Myeloid cells, Tumor-associated macrophages, Multiplex immunohistochemistry

## Abstract

**Background:**

Immunotherapy has demonstrated a limited clinical efficacy in approximately 5% of cholangiocarcinoma. The main challenges for an effective immunotherapy response in cholangiocarcinoma arise from the tumor microenvironment, which is poorly understood.

**Methods:**

For a comprehensive analysis of the tumor microenvironment in cholangiocarcinoma, we performed multiplex immunohistochemistry with two 15-marker immune panels and Nanostring assays for a comprehensive analysis of 104 surgically resected cholangiocarcinomas including intrahepatic, hilar, and distal cholangiocarcinoma. We also validated some key findings with a batch integration analysis of published single cell RNA sequencing data.

**Results:**

This study found that natural killer cells occupy the largest immune cell compartment in cholangiocarcinoma. Granzyme-B^+^CD8^+^ effector T cells are significantly associated with better overall survival in both intrahepatic and distal cholangiocarcinoma. Above 85% of intrahepatic cholangiocarcinomas with higher density of PD-1^−^EOMES^−^CD8^+^ effector T cells are associated with long-term survival. However, only the density of PD-1^−^EOMES^−^CD8^+^ T cells in the tumor areas, but not in the peripheries of the tumors, is prognostic. In all three cholangiocarcinoma subtypes, T regulator cells are significantly associated with a poor prognosis; however, M1 and M2 tumor-associated macrophages or PD-L1^+^ tumor-associated macrophage demonstrate different prognostic values. Combining PD-L1^+^ M1 or M2, PD-L1^−^ M1 or M2 tumor-associated macrophages, and T regulator cells to subgroup intrahepatic and distal cholangiocarcinoma, the prognosis is significantly better distinguished. Moreover, PD-L1^−^ M2 tumor-associated macrophages is associated with a good prognosis in intrahepatic and distal cholangiocarcinoma, suggesting this subtype of M2 tumor-associated macrophages may be antitumoral. Interestingly, lower densities of various types of immunosuppressive cells are associated with decreased infiltration of effector T cells in distal and hilar cholangiocarcinoma, but not in intrahepatic cholangiocarcinoma. In intrahepatic cholangiocarcinoma, PD-L1^+^ tumor-associated macrophages exert their immunosuppressive function likely through promoting T cell exhaustion.

**Conclusions:**

This study suggests that the densities of Granzyme-B^+^CD8^+^ effector T cells and non-exhausted PD-1^−^EOMES^−^CD8^+^ T cells and the PD-L1 status in the tumor-associated macrophages are prognostic makers in cholangiocarcinomas. The study also supports targeting PD-L1^+^ tumor-associated macrophages as the immunotherapy for cholangiocarcinoma.

**Supplementary Information:**

The online version contains supplementary material available at 10.1186/s13045-022-01253-z.

## Introduction

Globally, cholangiocarcinoma is one of the most common gastrointestinal malignancies [1]. In the United States and most of Europe, cholangiocarcinoma is associated with a high mortality rate in the setting of an increasing incidence in recent years [2]. Surgical resection remains the only curative modality for cholangiocarcinoma, however, is associated with a high recurrence [3]. Cholangiocarcinoma is categorized into intrahepatic and extrahepatic cholangiocarcinoma (ICC and ECC) according to its primary anatomic location [4]. Extrahepatic cholangiocarcinoma is further subdivided into hilar cholangiocarcinoma (HC) and distal cholangiocarcinoma (DCC), based on the proximity of tumor to the bifurcation of the common bile duct [5, 6]. Because neoplastic cells originate from different parts of the biliary tract, the tumor biology of ICC, HC, and DCC is anticipated to be different. Not surprisingly, genetic alterations in cholangiocarcinoma also differ significantly among the various anatomic types of cholangiocarcinoma [7]. Although molecular targeted therapies have been shown to be promising in treating patients with IDH mutations and FGFR2 gene fusions, only approximately 20% of cholangiocarcinoma harbor a targetable genetic alteration. Even with a targetable genetic alteration, only a fraction of these cholangiocarcinomas respond to matched targeted therapies [8, 9]. While systemic therapy has proven benefits in those with unresectable and metastatic disease, the effectiveness of chemotherapy and durability of its response are still quite limited [10].

Immunotherapy is a promising and paradigm shifting therapeutic modality that has emerged in the last few years because of its ability to control malignant diseases such as melanoma or NSCLC, which failed conventional therapies [11–13]. However, immunotherapy including immune checkpoint inhibitors demonstrates a limited clinical efficacy and achieve durable response in approximately 5% among all cholangiocarcinoma patients and less than 20% among PD-L1 positive patients [14–16]. Many solid tumors including cholangiocarcinoma face similar challenges in how to overcome the resistance to immune checkpoint inhibitors. It is now recognized that such challenges arise primarily from the immunosuppressive tumor microenvironment (TME) [17]. However, our understanding of the TME in cholangiocarcinoma and its role in propagating immune resistance to checkpoint inhibition as well as other immunotherapies is very limited.

Published studies on the TME in cholangiocarcinoma are mainly limited to PD-L1 expression and CD8 T-cell infiltration with significant variability and with only a few consensuses noted. First, membranous PD-L1 is infrequently expressed on the tumor cells in ICC, HC, or DCC, but is more frequently expressed on stromal cells such as macrophages [18–23]. Second, PD-L1 expression on tumor cells correlates with poor survival of ICC and ECC [19, 22, 24]. Some studies also demonstrated that PD-L1 expression on tumor cells correlates with increased CD8+ T cell infiltration [25, 26]. However, in these studies, the correlation between PD-L1 expression and poor survival was not supported [25, 26]. Nevertheless, one study showed that high PD-L1 expression and low T cell infiltration together are associated with poor survival [18]. Overall, published studies are limited in the types of immune cells assessed and lack correlative analyses between PD-L1 expression in stromal cells and patient outcomes. In addition, most published studies semi-quantify PD-L1 expression and tumor infiltrating lymphocytes (TILs).

In this study we performed, to our knowledge, the largest comprehensive biomarker analysis of the TME of cholangiocarcinoma employing our novel multiplex immunohistochemistry (IHC) technique. Through this study, we have created a broad atlas of tumor infiltrating immune cells in cholangiocarcinoma, providing the largest known resource of its kind for the research community of cholangiocarcinoma. Through this analysis we have revealed distinct immune signatures within the TME of ICC, HC, and DCC and their associations with clinical outcomes. The results of this study can inform the design of clinical trials of immunotherapy for cholangiocarcinoma.

## Methods

### Patients, specimens, and study approval

A total of 104 surgically resected cholangiocarcinoma formalin-fixed paraffin embedded (FFPE) specimens from consecutive patients who had undergone surgical resection at Johns Hopkins Hospital between January 1, 2005 and December 31, 2015 (Additional file 1: Table S1). Patients who had less than 3 years of follow-up were excluded. Patients whose FFPE blocks were of poor tissue quality were excluded. Under a Johns Hopkins Medical Institution Institutional Review Board approved protocol, FFPE blocks were obtained from the pathology archive and clinicopathologic information were obtained through chart review.

### Sequential IHC and image acquisition

The sequential staining-striping multiplex IHC protocol has been described previously [27]. In brief, slides with 5 μm of FFPE tissue sections were stained by hematoxylin, followed by whole-slide scanning using NanoZoomer (Hamamatsu). Then, following antigen retrieval by microwave treatment with the Antigen Retrieval Citra Plus Solution (Abcam), sequential IHC consisting of multiple iterative cycles of staining, scanning, and antibody and chromogen stripping was performed as described previously [27]. The concentration of primary antibodies and secondary antibody and incubation time of primary antibodies, horseradish peroxidase (HRP)-conjugated polymer, and chromogenic detection, respectively, are described in Additional file 1: Table S2. Two forms of negative control staining were performed at the end including a conventional negative control that was stained with 2.5% goat serum in PBS in the absence of primary antibodies and a second negative control that was the slide undergoing the complete antibody and chromogen stripping following the last cycle of staining.

In this study, we stained the first 20 slides with one panel of 22 biomarkers including both lymphoid and myeloid cell marker (Additional file 1: Table S2). The remaining slides were stained by two separate panels including one panel of 15 lymphoid cell markers and one panel of 15 myeloid cell markers. The panels depict CD8+ T cells, TH1, TH2, and TH17 T cells, regulatory T cells (TREG), B cells, and natural killer (NK) cells, tumor-associated macrophages (TAMs), immature (DC-SIGN+) versus mature (CD83+) dendritic cells (DCs), CD66b+ granulocytes (Gr), and tryptase+ mast cells.

### Image processing and analysis

The digitalized image processing and analyzing workflow included three steps: image co-registration, visualization, and quantitative image analysis (Additional file 1: Fig. S1). Digitized images were first co-registered using the CellProfiler v.2.1.1 pipeline as previously described [27]. Tumor areas were circled by pathologists (EDT, RAA). Up to five rectangular regions of interests (ROIs, approximately 5000*5000 pixels) in the vicinity of tumor epithelia were chosen to cover the entire tumor areas. EpCAM staining was used to maximally exclude tumor epithelia, large intratumoral blood vessels and the areas with tissues fallen off. Visualization was performed by converting co-registered images into individually pseudocolored single-marker images through the ImageJ software and the Aperio Image Scope software. For quantitative analysis, single cell segmentation and quantification of signals were performed using the CellProfiler v.2.1.1 pipeline as previously described [27], followed by image cytometry analysis with the FCS Express 6 Image Cytometry software (De Novo Software) (Additional file 1: Fig. S2). Immune cell subtypes were defined by multiple markers (Additional file 1: Table S3).

### Nanostring analysis

RNA was isolated from five 10 μm sections from FFPE samples, by using the Qiagen FFPE RNA kit, from 62 patients among above 104 patients including DCC, *n* = 33 and ICC, *n* = 29. The Nanostring assay showed a high correlation coefficient between fresh-frozen and FFPE samples previously [28]. In this study, highly degraded (> 60% Total) RNA samples would be excluded and subsequently replaced. RIN values were between 1.9 and 4.2 (median: 2.5) and considered to be superior among FFPE samples [28]. We used the human Pan-Cancer Immune Profiling Panel on a Nanostring Technologies nCounter platform to perform the immune gene expression profiling on the FFPE specimens. Raw data were normalized using the GenNorm algorithm and analyzed using the Nanostring nSolver advanced analysis software (version 2.0.115).

### Statistics

The density of each immune cell subtype was defined by calculating its percentage among all CD45+ cells in the tumor areas. The tumors whose density of a certain immune cell subtype is higher than the median were grouped in the “higher” density group for this immune cell subtype; and those whose density of this immune cell subtype is lower than the median were grouped in the “lower” density group. Kaplan–Meier curve and log-rank test were used to compare the distribution between the “higher” versus “lower” groups. Overall survival (OS) was calculated from the date of surgery to the date of death or to the date the patient was last known to be alive if death had not been reported at the time of analysis. OS longer than 3 years following surgical resection was considered to be a long-term survival in cholangiocarcinoma in previous studies [29, 30]. In addition, univariate and multi-variate logistic regression were used to model the association between OS (longer than 3 years vs. shorter than 3 years) and density of selected immune subtypes (“higher” vs. “lower”) controlling for other clinicopathological parameters. Clinicopathologic parameters whose odd ratio or 95% confidence interval in their correlation with OS (longer than 3 years vs. shorter than 3 years) is equal zero or infinity in the univariate or multivariate logistic regression were excluded from the model. It would be indicated in the results if tumors from all the patients with OS > 3 years or from all the patients with OS < 3 years have a “higher” immune cell density. All tests were two-sided and a *p*-value of < 0.05 was considered as statistically significant. All analyses were performed with R 2.14.0 and GraphPad Prism 8.0.2.

## Results

### Distinct patterns of immune cell subtypes in different types of cholangiocarcinoma

**Fig. 1:**
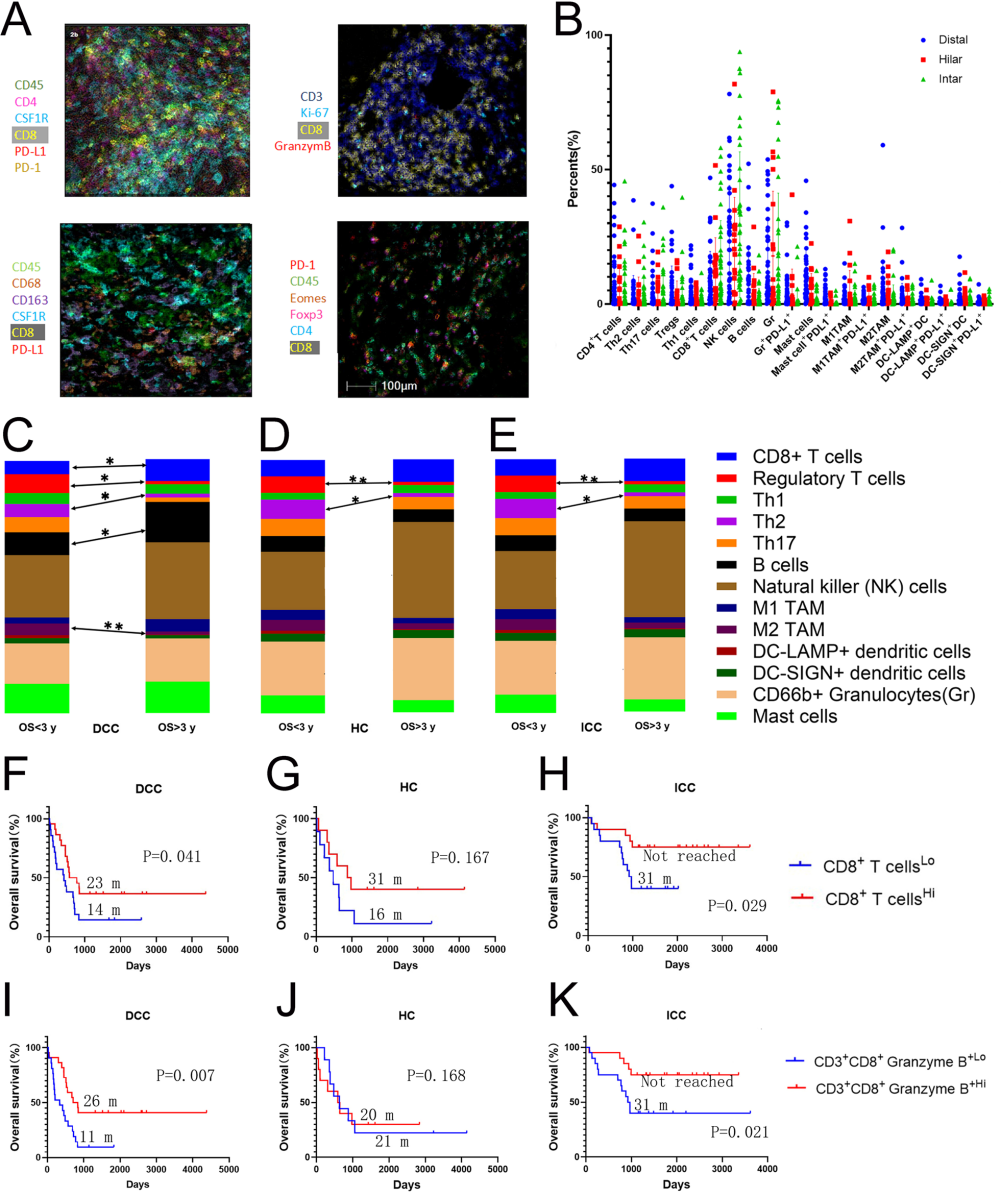
Multiplex immunohistochemistry of cholangiocarcinomas. **A** Overlaid multiple images stained with immune cell markers with pseudocolors and selected immune cell subtypes defined by multiple myeloid cell marker were shown. **B** Percentages of immune cell subtypes among CD45^+^ cells. **C**–**E** Immune cell composition according to the average percentages of each type of immune cells among CD45^+^ cells in three different types of cholangiocarcinoma divided into the group of OS < 3 years and the group of OS > 3 years. The percentages of each cell type were compared between the groups of OS < 3 years and OS > 3 years by *t* test. *p < 0.05; **p < 0.01. **F**–**K** Kaplan–Meier curves of overall survival of patients whose tumors are grouped by higher density versus lower density of CD8^+^ T cells or CD8^+^granzyme B^+^ T cell subtypes as indicated. Log rank tests were done with *p* values indicated. DCC, distal cholangiocarcinoma; HC, hilar cholangiocarcinoma; ICC, intrahepatic cholangiocarcinoma

We collected 104 cases of surgically resected cholangiocarcinomas including 44 ICCs, 20 HCs, and 40 DCCs (Additional file 1: Table S1) which were examined by a recently established sequential staining and stripping multiplex IHC technique [27] (Fig. 1A, Additional file 1: Fig. S1) with two panels of immune cell biomarkers including one panel of primarily lymphoid cell markers and one panel of primarily myeloid cell markers (Additional file 1: Table S2). A list of lymphoid and myeloid cell subtypes were defined by multiple markers and quantified (Additional file 1: Table S3). The cells that are considered positive for every marker are gated in reference to the cytometry of the negative control staining (Additional file 1: Fig. S2). Cells that are marked by pan-leucocyte marker, CD45, within the same size of tumor area appear to be similar among all patients. Therefore, the density of each immune cell subtype was calculated as their percentage of total CD45^+^ cells within the tumor areas (Fig. 1A). Compositions of immune cell subtypes within CD45^+^ leukocytes are different between cholangiocarcinoma subtypes and are different between patients whose OS is longer than 3 years (OS > 3 years) and those whose OS is shorter than 3 years (OS < 3 years) (Fig. 1C–E). The densities of mast cells, B cells, T helper 1 (Th1) CD4^+^ T cells, CD8^+^ T cells are significantly different between all three types of cholangiocarcinoma (Additional file 1: Fig. S3). Average densities of effector cells such as CD8+ T cells are increased in those with OS > 3 years versus OS < 3 years in all three types of cholangiocarcinoma with a statistically significant increase in T cells in DCC (Fig. 1C–E). The average density of B cells is also significantly increased in DCC with OS > 3 years versus OS < 3 years. Interestingly, the largest compartment in all three types of cholangiocarcinoma is occupied by natural killer (NK) cells. Immunosuppressive T helper (Th) cells including Th2 and T regulatory cells (Treg) are consistently and significantly decreased in all three types of cholangiocarcinoma with OS > 3 years versus OS < 3 years. Pro-tumoral M2 macrophages are decreased in all three types of cholangiocarcinoma with OS > 3 years versus OS < 3 years; and this decrease is statistically significant in DCC. These results suggest that both effector cells and immunosuppressive cells are potentially prognostic in cholangiocarcinoma.

### ***Infiltration of CD8***^+^***T cells is associated with better survival in cholangiocarcinoma***

We thus explored the prognostic values of the major immune subtypes. In all three types of cholangiocarcinoma, higher densities of CD8^+^ T cells are associated with better overall survival (OS) (Fig. 1F–H and Table 1). In DCC and ICC, CD8 infiltration is significantly associated with better OS (*p* = 0.04 and *p* = 0.029, respectively; Fig. 1F, H). Among ICC patients whose tumors had higher densities of CD8^+^ T cells, approximately 75% had a long-term survival (> 3 years) following the surgical resection. By contrast, among DCC patients whose tumors had higher densities of CD8^+^ T cells, only approximately 40% achieved a long-term survival. CD8^+^granzyme B^+^ cells were examined to evaluate the effector cell phenotype of CD8^+^ T cells (Fig. 1I–K). As anticipated, higher density of CD8^+^granzyme B^+^ cells is significantly associated with better OS in both DCC (*p* = 0.007) and ICC (0.021) (Fig. 1I, K). However, CD8^+^ T cells or CD8^+^granzyme B^+^ T cells are not associated with OS in HC likely due to the limited sample size (Fig. 1G, J). B cells were also examined and found to have a significant prognostic value in ICC, but less significant in DCC, and not significant in HC. More specifically, in ICC, higher density of B cells is significantly associated with better OS in both the Kaplan–Meier analysis (*p* = 0.004) and multivariate analysis (*p* = 0.004) (Additional file 1: Fig. S4 and Additional file 1: Table S4–S6). This result suggests that B cells may have antitumor activities in ICC and DCC. On another hand, NK cell infiltration also does not appear to have an association with clinical outcomes in all three types of cholangiocarcinoma (Additional file 1: Fig. S4).

**Table 1:** Summary of univariate and multivariate logistic analysis of the association between the density of effector T cells or tumor associated macrophages (TAM) and OS < 3 years

BTC type	Immune cell subtypes	Univariate Analysis			Multivariate analysis		
		Odds ratio	95% CI	*p* value	Odds ratio	95% CI	*p* value
DCC	CD8^+^ T cells: High versus Low	0.276	0.062, 1.233	0.092	0.238	0.049, 1.158	0.075
HC	CD8^+^ T cells: High versus Low	0.167	0.532, 67.64	0.147	0.181	0.015, 2.24	0.183
ICC	CD8^+^ T cells: High versus Low	0.222	0.058, 0.858	0.029	0.218	0.038, 1.247	0.087
DCC	CD8^+^ Granzyme B^+^ T cells: High versus Low	0.144	0.027, 0.778	0.024	0.114	0.122, 2.062	0.02
HC	CD8^+^ Granzyme B^+^ T cells: High versus Low	0.583	0.075, 4.562	0.608	0.287	0.017, 4.835	0.386
ICC	CD8^+^ Granzyme B^+^ T cells: High versus low	0.222	0.058, 0.858	0.029	0.269	0.05, 1.435	0.124
DCC	CD8^+^EOMES^−^PD-1^−^ T cells: High versus Low	0.476	0.117, 1.944	0.301	0.501	0.122, 2.062	0.338
HC	CD8^+^ EOMES^−^PD-1^−^ T cells: High versus Low	0	0, Infinity*	0.995	0	0, Infinity*	0.996
ICC	CD8^+^EOMES^−^PD-1^−^ T cells: High versus low	0.076	0.016,0.358	0.001	0.049	0.006, 0.433	0.007
DCC	PD-L1^+^ CSF-1R^+^ TAM: High versus Low	6.923	1.285, 37.287	0.024	8.173	1.402, 47.635	0.019
HC	PD-L1^+^ CSF-1R^+^ TAM: High versus Low	1.714	0.219, 13.406	0.608	5.583	0.261, 119.634	0.271
ICC	PD-L1^+^ CSF-1R^+^ TAM: High versus Low	7.429	1.778, 31.04	0.006	10.152	1.658,62.177	0.012
DCC	M1 TAM: High versus Low	0.476	0.117, 1.944	0.301	0.449	0.105, 1.927	0.281
HC	M1 TAM: High versus Low	0.583	0.075, 4.562	0.608	0.527	0.062, 4.449	0.556
ICC	M1 TAM: High versus Low	0.222	0.058, 0.858	0.029	0.208	0.039, 1.104	0.065
DCC	PD-L1^+^ M1 TAM: High versus Low	6.923	1.285, 37.287	0.024	7.07	1.303, 38.349	0.023
HC	PD-L1^+^ M1 TAM: High versus Low	1.714	0.219, 13.406	0.608	0.665	0.041, 10.784	0.774
ICC	PD-L1^+^ M1 TAM: High versus Low	2.852	0.777,10.467	0.114	6.166	0.875,43.46	0.068
DCC	M2 TAM: High versus Low	6.923	1.285, 37.287	0.024	7.473	1.365, 40.907	0.02
HC	M2 TAM: High versus Low	6	0.532, 67.64	0.147	5.194	0.213, 126.625	0.312
ICC	M2 TAM: High versus Low	1.227	0.35,4.307	0.749	2.35	0.467,11.831	0.3
DCC	PD-L1^+^ M2 TAM: High versus Low	3 × 10^8^	0, Infinity*	0.993	3 × 10^8^	0, Infinity*	0.993
HC	PD-L1^+^ M2 TAM: High versus Low	0.583	0.075, 4.562	0.608	0.244	0.012, 5.12	0.364
ICC	PD-L1^+^ M2 TAM: High versus Low	7.429	1.778, 31.04	0.006	10.812	1.63, 71.735	0.014

*Tumors from all the patients with OS > 3 years or from all the patients with OS < 3 years have a “higher” immune cell density; BTC, biliary tract cancer; DCC, distal cholangiocarcinoma; HC, hilar cholangiocarcinoma; ICC, intrahepatic cholangiocarcinoma

### ***Distinct patterns and prognostic values of CD4***^+^***T cells in different type of cholangiocarcinoma***

**Fig. 2:**
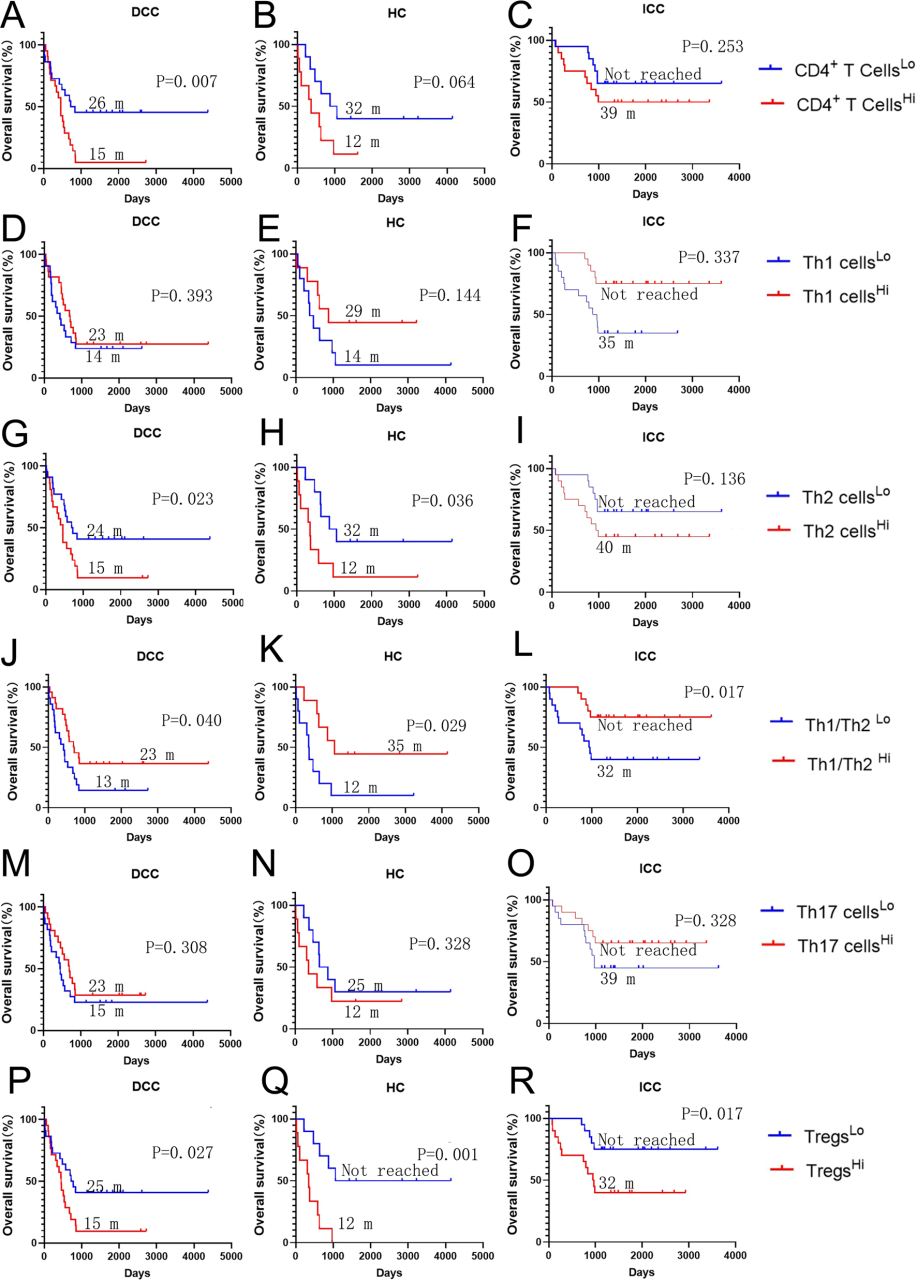
The role of CD4+ cells in the overall survival of cholangiocarcinoma. Kaplan–Meier curves of overall survival (OS) of patients whose tumors are grouped by higher density versus lower density of CD4^+^ T cells or CD4^+^ T helper (Th) cell subtypes as indicated. The correlation of the density of general CD4^+^ T cells (**A**–**C**), Th1 cells (**D**–**F**), Th2 cells (**G**–**I**), the ratio of Th1/Th2 cells (**J**–**L**), the density of Th17 cells (**M**–**O**), and Treg cells (**P**–**R**), respectively, with OS. Log rank tests were performed with *p* values as indicated. DCC, distal cholangiocarcinoma; HC, hilar cholangiocarcinoma; ICC, intrahepatic cholangiocarcinoma

The role of CD4^+^ T cells appears to be different among ICC, HC, and DCC (Fig. 2). In ICC, there is no significant difference in OS between tumors with higher and lower densities of CD4^+^ T cells. By contrast, higher density of CD4^+^ T cells is associated with significantly poorer OS in DCC (*p* = 0.007) and with a trend (*p* = 0.064) towards poorer OS in HC (Fig. 2). Examination of CD4^+^ T cell subtypes, such as CD3^+^CD4^+^Foxp3^+^ Tregs, revealed some clues as to why CD4^+^ T cells in DCC and HC are negative prognostic factors. In all three types of cholangiocarcinoma, Tregs, a dominant CD4^+^ T cell subtype, are strongly associated with poorer prognosis (DCC: *p*  = 0.027; HC: *p* = 0.001; ICC: *p* = 0.017) (Fig. 2). Although neither Th1 nor Th2 cells are associated with survival in ICC, a higher ratio of Th1/Th2 is significantly associated with better OS in this type of cholangiocarcinoma (*p* = 0.017) (Fig. 2). These results suggest that Tregs contribute the most to the prognostic value of CD4^+^ cells infiltrating ICC. By contrast, higher densities of Th2 cells are significantly associated with poorer survival in DCC (*p*  = 0.023) whereas Th1 cells are not associated with the survival in DCC. Therefore, both Th2 cells and Tregs contribute to the poor prognosis associated with CD4^+^ T cells infiltrating in DCC. In HC, both a lower density of Th2 cells (*p* = 0.036) and a higher Th1/Th2 ratio (*p* = 0.029) is significantly associated with the survival (Fig. 2). Therefore, all three subtypes of cholangiocarcinoma appear to have distinct Th1/Th2 patterns in their respective TMEs.

### ***CD8***^+^***T cells that lack expression of both PD-1 and EOMES have a strong prognostic value in predicting long-term survival in intrahepatic cholangiocarcinoma***

**Fig. 3:**
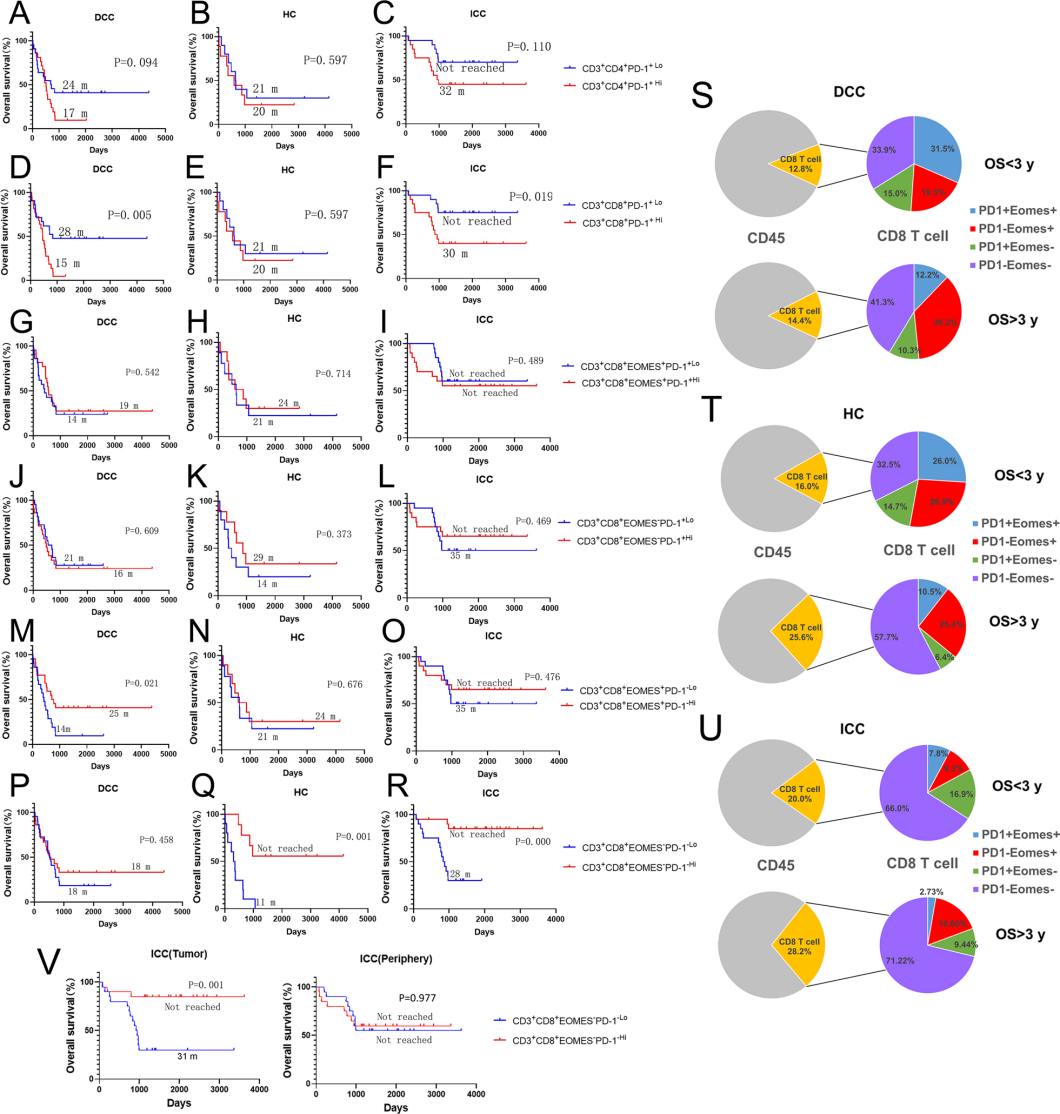
The role of T cell exhaustion markers in the overall survival of cholangiocarcinoma. A-R and V, Kaplan–Meier curves of overall survival (OS) of patients whose tumors are grouped by higher density versus lower density of exhausted T cell subtypes as indicated. Log rank tests were performed with *p* values as indicated. The correlation of the density of CD4^+^PD-1+ T-cells (**A**–**C**), CD8^+^PD-1^+^ T-cells (**D**–**F**), CD8^+^EOMES^+^PD-1^+^ T-cells (**G**–**I**), CD8^+^EOMES^−^PD-1^+^ T-cells (**J**–**L**), CD8^+^EOMES^+^PD-1^−^ T-cells (**M**–**O**), and CD8^+^EOMES^−^PD-1^−^ T-cells (**P**–**R**) with OS. (**S**–**U**) Percentages of CD8^+^ T cells among CD45^+^ T cells were shown in the pie graphs; and CD8^+^ T cells were further divided according to the status of PD-1 and EOMES as shown in the pie graphs. V Kaplan–Meier curves of overall survival of patients whose tumors are grouped by higher density versus lower density of CD3^+^CD8^+^EOMES^−^PD-1^−^ T cells in the tumor areas (left panel) and in the peripheries of the tumors (right panel), respectively. DCC, distal cholangiocarcinoma; HC, hilar cholangiocarcinoma; ICC, intrahepatic cholangiocarcinoma

PD-1^+^ T cells also appear to have varying distribution patterns and prognostic values in different types of cholangiocarcinoma (Fig. 3). Lower density of CD4^+^PD-1^+^ T cells is shown to have a trend associated with better OS in DCC and ICC (Fig. 3A–C). By contrast, lower density of CD8^+^PD-1^+^ T cells is significantly associated with better OS in both DCC (*p* = 0.005) and ICC (*p* = 0.019), but not in HC (*p* = 0.597) (Fig. 3D–F). Consistently, in all three types of cholangiocarcinoma, those with OS > 3 years have lower percentages of PD-1^+^ cells among CD8^+^ T cells than those with OS < 3 years (Fig. 3S–U). This result confirmed that PD-1 is a factor for poor prognosis in ICC and DCC. However, when CD8^+^PD-1^+^ cells are divided into CD8^+^PD-1^+^EOMES^+^ and CD8^+^PD-1^+^EOMES^−^ cells, neither subtype of CD8^+^PD-1^+^ cells is associated with OS in any of the three types of cholangiocarcinoma (Fig. 3G–R). By contrast, higher density of CD8^+^PD-1^−^EOMES^+^ T cells is significantly associated with better OS in DCC (*p*  = 0.021), likely because CD8^+^ T cells in general are associated with better OS in DCC (Fig. 1). Therefore, PD-1 and EOMES represent two independent exhaustion pathways in all three types of cholangiocarcinoma; and other T cell exhaustion markers [31] including LAG3, TIM3, TOX were thus investigated below. In ICC, EOMES^+^CD8^+^ T cells do appear to distribute similarly as LAG3^+^ or TIM3^+^ CD8^+^ T cells particularly in the tumors with possibly a more immunosuppressive TME according to the single cell RNA sequencing analysis (below Additional file 1: Fig. S17), suggesting that EOMES is a T cell exhaustion marker as LAG3 and TIM3 in ICC. Further supporting this notion, a higher density of PD-1/EOMES-double negative CD8^+^ T cells (CD8^+^PD-1^−^EOMES^−^) T cells is significantly associated with better OS in both HC (*p* = 0.001) and ICC (*p* = 0.0001) (Fig. 3P–R). Interestingly, CD8^+^PD-1^−^EOMES^−^ T cells appear to be a dominant subtypes of CD8+ T cells in both HC and ICC, particularly in those with OS > 3 years (Fig. 3T, U). The function of this CD8+ T cell subtype remains to be investigated.

To understand which effector cell parameter(s) may predict long-term survival, univariate and multivariate logistic regression were used to model the association between OS (> 3 years vs. < 3 years) and density of selected immune subtypes (“high” vs. “low”) controlling for other clinicopathological parameters (Table 1 and Additional file 1: Table S4–S18). CD8^+^PD-1^−^EOMES^−^ T cells, which lack expression of both PD-1 and EOMES, appear to be the best immune parameter among all examined which may potentially predict long-term survival in ICC (*p* = 0.007) (Fig. 3 and Table 1). More than 85% of ICCs patients whose tumors had higher density of CD8^+^PD-1^−^EOMES^−^ T cells had a long-term survival (> 3 years) following surgical resection. This CD8+ T cell subtype, which is associated with good prognosis, is noteworthy of being studied further in all three types of cholangiocarcinoma. By contrast, the density of CD8^+^Granzyme B^+^ T cells is a stronger predictor of long-term survival (*p* = 0.02) than that of CD8^+^PD-1^−^EOMES^−^ T cells in DCC (Fig. 1 and Table 1).

We further divided the ROIs into the tumor areas containing neoplasm cells and the peripheries outside the tumor areas and re-assessed the density of each lymphoid cell subtype in the tumor areas compared to the peripheries of the same cholangiocarcinoma samples. We found that the densities of NK cells, B cells, Th17 cells, CD3^+^CD8^+^EOMES^+^PD-1^−^ T cells, and CD3^+^CD8^+^EOMES^−^PD-1^+^ T cells, respectively, were significantly decreased in the peripheries compared to the tumor areas in DCC, but not in ICC (Additional file 1: Figs. S5, S6). We also found that the densities of NK cells, Th2 cells, CD8^+^Granzyme B^+^ T cells, and CD3^+^CD8^+^EOMES^−^PD-1^−^ T cells, respectively, were significantly decreased in the peripheries compared to the tumor areas in ICC, but not DCC (Additional file 1: Figs. S5, S6). The survival association of CD3^+^CD8^+^EOMES^−^PD-1^−^ T cells, which were demonstrated above to be the best immune parameter predictive for long-term survival in ICC, is different between tumor areas and peripheries. In the tumor areas, but not in the peripheries of the tumors, the higher density of this non-exhausted T cell subpopulation is associated significantly with better OS (*p* = 0.001) (Fig. 3V). Thus, it is conceivable that CD3^+^CD8^+^EOMES^−^PD-1^−^ T cells that infiltrate within the tumor areas may play a stronger role in antitumor immune response in ICC than those in the peripheries of the tumors or that a stronger antitumor immune response would be elicited only when this non-exhausted subtype of CD8^+^ T cells have infiltrated into the tumor areas.

### M1 and M2 macrophages play a distinct role in the prognosis of intra-hepatic and distal cholangiocarcinoma

**Fig. 4:**
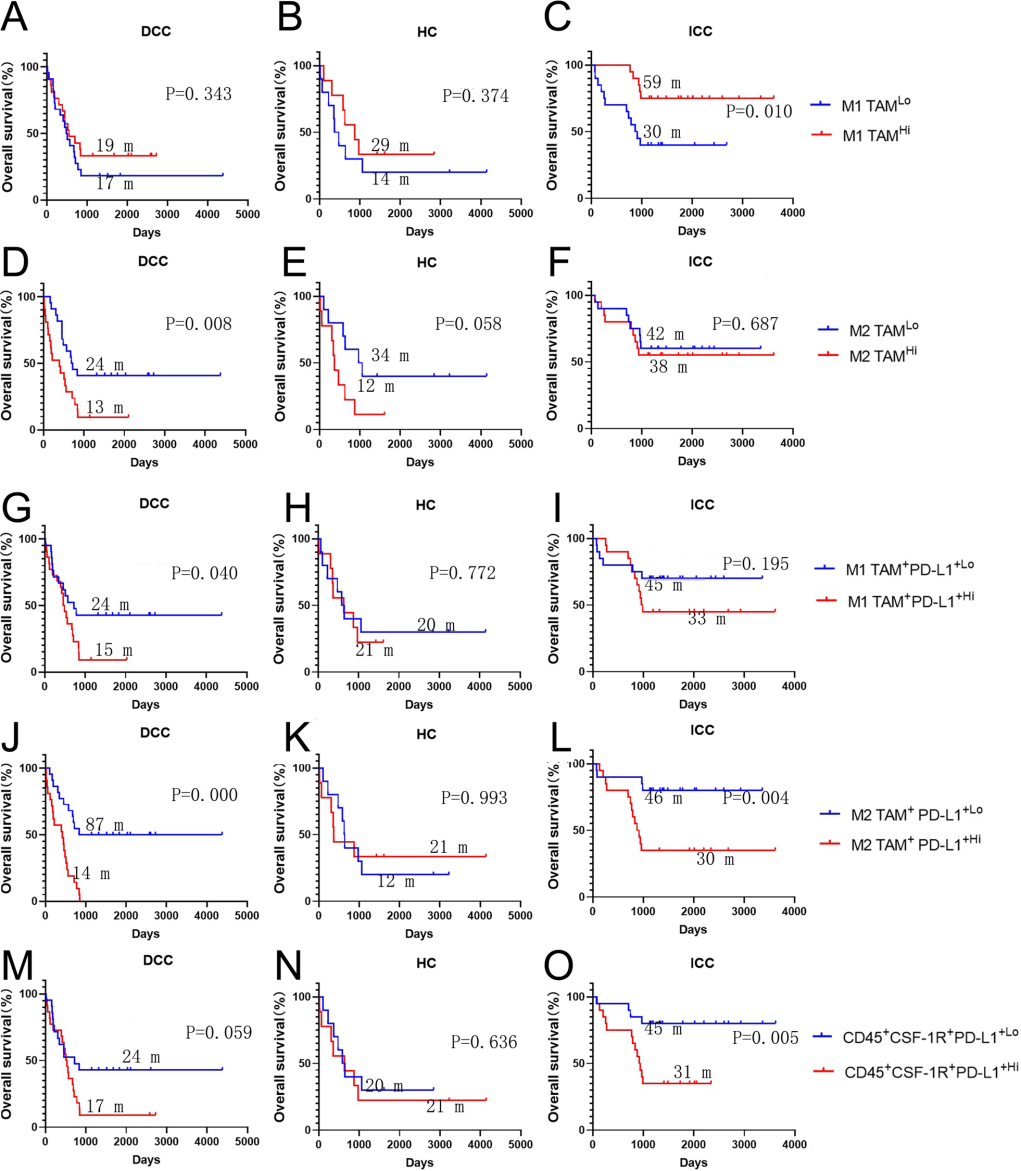
The role of tumor associated macrophages (TAM) or PD-L1+ TAM in the overall survival of cholangiocarcinoma. Kaplan–Meier curves of overall survival (OS) of patients whose tumors are grouped by higher density versus lower density of different types of TAMs or PD-L1^+^ TAM as indicated. The correlation of the density of M1-TAM (**A**–**C**), M2-TAM (**D**–**F**), PD-L1+ M1-TAM (**G**–**I**), PD-L1^+^M2-TAM (**J**–**L**), and PD-L1^+^CSF-1R^+^ myeloid cells (**M**–**O**) with OS. Log rank tests were performed with *p* values as indicated. DCC, distal cholangiocarcinoma; HC, hilar cholangiocarcinoma; ICC, intrahepatic cholangiocarcinoma

Next, we examined myeloid cell subtypes in all three types of cholangiocarcinomas. We did not observe a significant association between prognosis and with neutrophils, dendritic cells, or mast cell densities in any of the three types of cholangiocarcinoma (Additional file 1: Fig. S5). By contrast, densities of tumor associated macrophages (TAM) are associated with survival in all three types of cholangiocarinoma. Higher density of anti-tumoral M1-like TAMs (CD45^+^CD3^−^CD20^−^CD56^−^CD66b^−^Tryptase^−^CSF1R^+^CD68^+^CD163^−^) is significantly associated with better OS in ICC (*p* = 0.01) whereas pro-tumoral M2-like TAM (CD45^+^CD3^−^CD20^−^CD56^−^CD66b^−^Tryptase^−^CSF1R^+^CD68^+^CD163^+^) is not associated with OS in ICC (Fig. 4A–F). By contrast, higher density of M2 TAMs is significantly associated with poorer OS in DCC (*p* = 0.008), whereas M1 TAMs is not associated with OS in DCC (Fig. 4A–F). In HC, neither M1 TAMs nor M2 TAMs showed a statistically significant association with OS, although there was a strong trend (*p* = 0.058) towards poorer OS in HC with higher density of M2 TAMs. Taken together, these results also suggested that TAMs play a more prominent role in the TME of cholangiocarcinoma than any other myeloid subtype and should be considered as a major target for the immunotherapy of cholangiocarcinoma.

### ***PD-L1 plays an important role in the function of TAMs and CSF-1R***^+^***myeloid cells in intrahepatic and distal cholangiocarcinoma, but not in hilar cholangiocarcinoma***

As previously reported, PD-L1 is mainly expressed in myeloid cells in the TME of all three types of cholangiocarcinoma [18–23]. Among different myeloid cell subtypes, PD-L1^+^ (PD-L1 positive) neutrophils have the largest quantity. However, only a small percentage of neutrophils are PD-L1^+^ (Fig. 1B). Among the different myeloid subtypes examined, TAMs appear to have the highest percentages of cells expressing PD-L1. In both ICC and DCC, higher density of PD-L1+  M1 TAMs (CD45^+^CD3^−^CD20^−^CD56^−^CD66b^−^Tryptase^−^CSF1R^+^CD68^+^CD163^−^PD-L1^+^) or PD-L1+ M2 TAMs (CD45^+^CD3^−^CD20^−^CD56^−^CD66b^−^Tryptase^−^CSF1R^+^CD68^+^CD163^+^PD-L1^+^) is associated with poorer survival even though M1 TAMs in general are not associated with poor survival (Fig. 4G–I). PD-L1^+^ neutrophils are not associated with OS in all three types of cholangiocarcinoma (Additional file 1: Fig. S6). However, of other myeloid cell subtypes including dendritic cells and mast cells, either PD-L1 expression, higher density of PD-L1^+^ cells, or both, is associated with poorer OS and supports targeting both PD-1/PD-L1 and myeloid cells in DCC and ICC. As CSF-1R inhibitors are tested as a myeloid cell targeting agent in clinical trials, we examined the survival outcome association of CD45^+^CD3^−^CSF-1R^+^PD-L1^+^ cells, which include both PD-L1^+^ TAM and dendritic cells. The results confirmed that higher density of CD45^+^CD3^−^CSF-1R^+^PD-L1^+^ cells is associated with poorer OS in both ICC and DCC, but not in HC (Fig. 4M–O), supporting either PD-1/PD-L1, CSF1-R, or both in a combination as a therapeutic target for ICC and DCC. This immune cell parameter is also the strongest prognostic feature of poor survival in DCC and ICC among all the myeloid cell subtypes tested according to univariate and multi-variate analyses (Table 1 and Additional file 1: Tables S19–S33).

We further divided the ROIs into the tumor areas containing neoplasm cells and the peripheries outside the tumor areas and re-assessed the density of each myeloid cell subtype in the tumor areas compared to the peripheries of the same cholangiocarcinoma samples. We found that only the density of above described CD45^+^CD3^−^CSF-1R^+^PD-L1^+^ cells was significantly decreased in the peripheries compared to the tumor areas in DCC (Additional file 1: Fig. S9). In the tumor areas, the higher density of this myeloid cell subpopulation appears to be associated with poorer OS in a stronger trend (*p* = 0.064) than that in the peripheries (Additional file 1: Fig. S6). Thus, it is conceivable that CD45^+^CD3^−^CSF-1R^+^PD-L1^+^ cells that infiltrate within the tumor areas may play a stronger role in suppressing antitumor immune response in DCC than those in the peripheries of the tumors.

### Combined immune biomarkers divide DCC and ICC into prognostically more distinguished subgroups

**Fig. 5:**
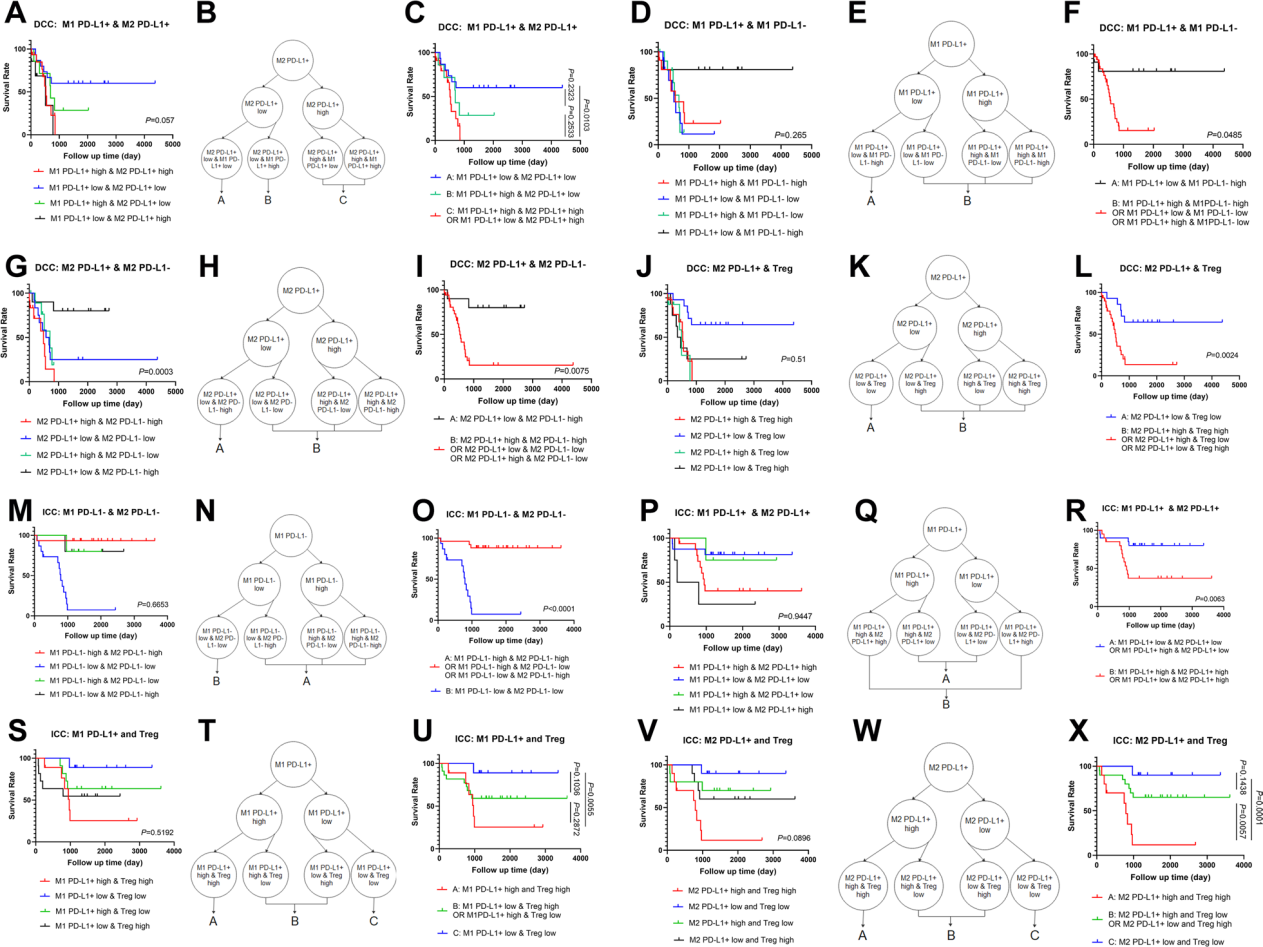
Subgrouping of DCC and ICC with combined biomarkers. **A**, **D**, **G**, **J**, **M**, **P**, **S**, **V** Kaplan–Meier curves of overall survival of patients whose tumors are subgrouped by higher density versus lower density of two immune cell subtypes including PD-L1^+^ or PD-L1^−^, M1 or M2 TAM and/or Treg as indicated. **B**, **E**, **H**, **K**, **N**, **Q**, **T**, **W** Schematic recombination of subgroups defined by two immune cell subtypes. **C**, **F**, **I**, **L**, **O**, **R**, **U**, **X** Kaplan–Meier curves of overall survival of patients whose tumors are re-grouped by following the schematic recombination of subgroups defined by two immune cell subtypes as indicated

Above results also suggest that TAM is a heterogeneous population and simply using PD-L1 expression to distinguish the prognostic values of TAM is not sufficient. Therefore, we further took advantage of our multiplex IHC approach by using PD-L1^+^ M1 TAM and PD-L1^+^ M2 TAM as biomarkers simultaneously to divide patients into four groups (Fig. 5A). In DCC, we found that, if the density of PD-L1^+^ M2 TAM is high, regardless the status of PD-L1^+^ M1 TAM, the prognosis is poor. If the density of PD-L1^+^ M1 TAM is low and that of PD-L1^+^ M2 is low, the prognosis is the best among the four groups. If the density of PD-L1^+^ M1 TAM is high, but that of PD-L1^+^ M2 TAM is low, the prognosis is intermediate (Fig. 5A). Thus, the group of high PD-L1^+^ M2 TAM and low PD-L1^+^ M1 TAM can be combined with the group of high PD-L1^+^ M2 TAM and high PD-L1^+^ M1 TAM (Fig. 5B). After subgrouping DCC into three groups, their prognosis is more distinguishable (Fig. 5C). Nevertheless, such a result suggests that targeting DCC with one immunotherapy strategy in absence of biomarker selection may not be successful.

Our multiplex IHC approach also allows us to quantify PD-L1^−^ (PD-L1 negative) TAM, including PD-L1^−^ M2 TAM, which may still have immunosuppressive functions. We found that, if we combine both PD-L1^+^ M1 TAM and PD-L1^−^ M1 TAM as biomarkers simultaneously to divide patients into four groups (Fig. 5D–F), the prognosis of DCC can be the best distinguished (*p* = 0.0485). Approximately 80% of DCCs with a low density of PD-L1^+^ M1 TAM but a high density of PD-L1^−^ M1 TAM demonstrate a long-term survival. Otherwise, DCC would have a very poor prognosis. Surprisingly, using a similar approach, we found that, if we use both PD-L1^+^ M2 TAM and PD-L1^−^ M2 TAM as a combined biomarker (Fig. 5G–I), approximately 80% of DCCs with low density of PD-L1^+^ M2 TAM and high density of PD-L1^−^ M2 TAM demonstrate a long-term survival. This result confirms that PD-L1^+^ M2 TAM is pro-tumoral, however, also suggests that PD-L1^−^ M2 TAM is associated with a good prognosis in DCC and thus may have an antitumor function. The association of the PD-L1^−^ M2 TAM with longer survival also supports the recent notion that the polarized classification of TAMs may not be appropriate [32] and the TAMs are highly plastic [33, 34].

We did not observe an advantage of the above combined biomarker strategy in subgrouping ICC with both PD-L1^+^ TAM and PD-L1^−^ TAM together. Next, we combined PD-L1^−^ M1 TAM and PD-L1^−^ M2 TAM as biomarkers simultaneously to divide patients into four groups (Fig. 5M). We did not observe an advantage of this combined biomarker strategy in subgrouping DDC; however, we found in ICC that, if both densities of PD-L1^−^ M1 TAM and PD-L1^−^ M2 TAM are low, the prognosis of ICC is poor (Fig. 5M). Excluding those with low PD-L1^−^ M1 TAM and low PD-L1^−^ M2 TAM, the remaining ICC all appear to have a good prognosis with essentially a cure after surgery (Fig. 5N, O). Here, similar to DCC, high density of PD-L1^−^ M2 TAM is also associated with a good prognosis albeit in a subgroup of ICC; however, different from DCC, high density of PD-L1^−^ M2 TAM or high density of PD-L1^−^ M1 TAM, not necessarily both together, is associated with a good prognosis in ICC. In addition, this PD-L1^−^ M1/PD-L1^−^ M2 TAM combination biomarker stratification strategy surpasses all the immune biomarkers examined above in distinguishing the prognosis of ICC (*p* = 0.0001).

Next, we combined PD-L1^+^ M1 TAM and PD-L1^+^ M2 TAM as biomarkers simultaneously to divide patients into four groups in ICC (Fig. 5P**)**. We found that the poor prognosis in ICC is solely determined by the high density of PD-L1^+^ M2 TAM (Fig. 5P). After merging the four groups into two groups (Fig. 5Q), this approach (Fig. 5R), which was taken independently, confirmed the result in Fig. 4I.

We also combined PD-L1^+^ TAM and Treg as biomarkers simultaneously to divide patients into four groups in DCC and in ICC, respectively (Fig. 5J, S, V). We did find that using this combined biomarker strategy in DCC with both PD-L1^+^ M2 TAM and Treg (Fig. 5J–L) is superior over using Treg alone by dividing the patients into two prognostically more distinguished groups (Fig. 2P), suggesting that PD-L1^+^ M2 TAM and Treg play independent roles in DCC. In ICC, combining Treg with either PD-L1^+^ M1 TAM or PD-L1^+^ M2 TAM (Fig. 5S, V), respectively, as immune parameters identifies one subgroup of ICC with approximately 90% being long-term survivors and another subgroup with more than 90% having a dismal prognosis (Fig. 5U, X). However, this combined biomarker strategy also identifies a third subgroup with intermediate prognosis (Fig. 5U, X). Taken together, these results support targeting both PD-L1^+^ TAM and Treg in both ICC and DCC. We did not use the combined biomarker strategy to re-analyze the multiplex IHC data of HC due to the small sample size. Nevertheless, such a strategy should be applied to HC in the future studies.

### Lower density of immunosuppressive cells is associated with a decreasing infiltration of effector T cells in distal and hilar cholangiocarcinoma, but not in intrahepatic cholangiocarcinoma

**Fig. 6:**
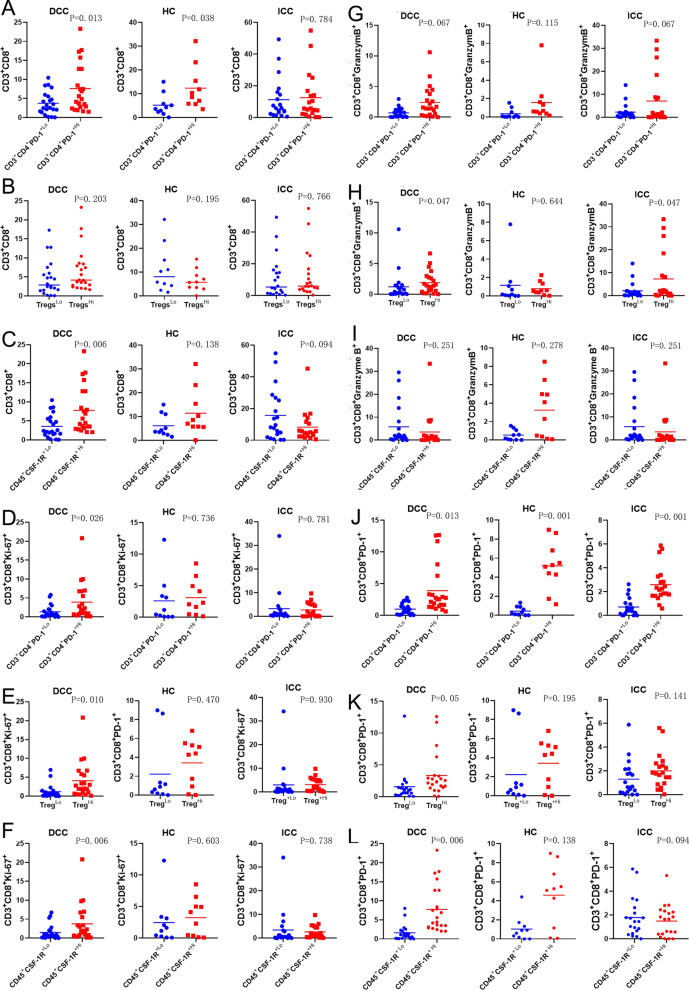
Comparison of various effector T cell subtypes between cholangiocarcinomas with higher versus lower density of regulatory immune cells. Tumors are subgrouped by higher density versus lower density of two immune cell subgroups of CD4^+^PD-1^+^ T cells in **A**, **D**, **G**, **J**, Tregs in **B**, **E**, **H**, **K**, CSF1-R^+^ myeloid cells in **C**, **I**, **F**, **L**. The density of CD8^+^ T-cells (**A-C**), CD8^+^Ki67^+^ T-cells (**D-F**), CD8^+^Granzyme B^+^ T-cells (**G**–**I**), CD8^+^PD-1^+^ T-cells (**J**–**L**) was compared between the two subgroups. *t* tests were performed with *p* value indicated. DCC, distal cholangiocarcinoma; HC, hilar cholangiocarcinoma; ICC, intrahepatic cholangiocarcinoma

Next, we examined how regulatory immune cells including CD4^+^PD-1^+^ T cells, Tregs, neutrophils, M1 TAM, M2 TAM, and CSF-1R^+^ myeloid cells may influence the infiltration of effector T cells and their subtypes. To this end, we divided each type of cholangiocarcinoma into two groups in the same way as the above survival analyses according to whether the regulatory immune subtype densities are higher or lower than their respective medians and compared the effector T cells between two groups. We found that a lower density of CD4^+^PD-1^+^ T cells is associated with a lower density of CD8^+^PD-1^+^ T cells consistently in all three types of cholangiocarcinoma (Fig. 6). Interestingly, a lower density of Tregs and CSF-1R^+^ cells is associated with lower density of CD8^+^ T cells, CD8^+^Ki67^+^T cells, CD8^+^granzyme B^+^ T cells, or CD8^+^PD-1^+^ T cells in DCC (Fig. 6 and Additional file 1: Fig. S10); and these associations are either statistically significant or in a strong trend. Such associations appear to be less strong, but maintain overall a similar trend in HC. In addition, lower density of anti-tumoral M1 TAMs is also associated with lower density of CD8^+^ T cells, CD8^+^Ki67^+^ T cells, or CD8^+^granzyme B^+^ T cells in HC (Fig. 6 and Additional file 1: Fig. S11). By contrast, such associations do not appear to exist in ICC. Indeed, lower density of CSF-1R^+^ cells and higher density of Th17 cells in ICC is associated with higher density of CD8^+^ T cells (Fig. 6 and Additional file 1: Fig. S12). These results may not be fully interpretable due to overall limited sample sizes and would need to be further examined by a linear regression analysis with larger sample sizes. However, they suggest that a decreasing infiltration of multiple subtypes of immunosuppressive cells such as Tregs and CSF-1R^+^ cells could be associated with a decreasing infiltration of effector T cells in DCC and HC. The underlying mechanism for such associations requires further investigation and whether therapeutic strategies that would lead to depletion of Tregs or CSF-1R^+^ cells in DCC and HC would ultimately lead to a decrease of effector T cells remains an important question. Nevertheless, therapeutic strategies of targeting and leading to depletion of CSF-1R^+^ cells in ICC are anticipated to increase infiltration of effector T cells.

### ***Correlation between PD-L1***^+^***myeloid cells and effector T cells in different types of cholangiocarcinoma***

We also compared effector T cell densities between cholangiocarcinomas with higher and those with lower density of myeloid cell subgroups that express PD-L1. In contrast with published studies which show a positive correlation between PD-L1 expression and CD8^+^ T cells in semi-quantitative IHC analysis [25, 26], our results demonstrated that, for most of myeloid cell subgroups, effector T cell densities do not appear to be significantly different between tumors with higher and those with lower density of myeloid cell subgroups that express PD-L1. However, in all three types of cholangiocarcinoma, lower density of PD-L1^+^DC-LAMP^+^ mature dendritic cells or PD-L1^+^DC-SIGN^+^ immature dendritic cells is associated with lower density of CD8^+^PD-1^+^ T cells either significantly or in a strong trend (Additional file 1: Figs. S13–S15). Thus, these results suggest that targeted depletion of PD-L1^+^ dendritic cells would have a potential therapeutic benefit by decreasing primarily the PD-1^+^ subgroup of effector T cells in all three types of cholangiocarcinoma.

Interestingly, in ICC, lower density of PD-L1^+^ M2 TAM is significantly associated with higher density of CD8^+^PD-1^−^EOMES^−^ T cells, which, as shown above, is the best predictor of long-term survival in ICC (Additional file 1: Fig. S16). This finding further highlights important roles of the PD-L1^+^ M2 TAM and the non-exhausted CD8^+^PD-1^−^EOMES^−^ effector T cells in ICC.

### TAM in DCC is associated with immunosuppressive mechanisms

**Fig. 7:**
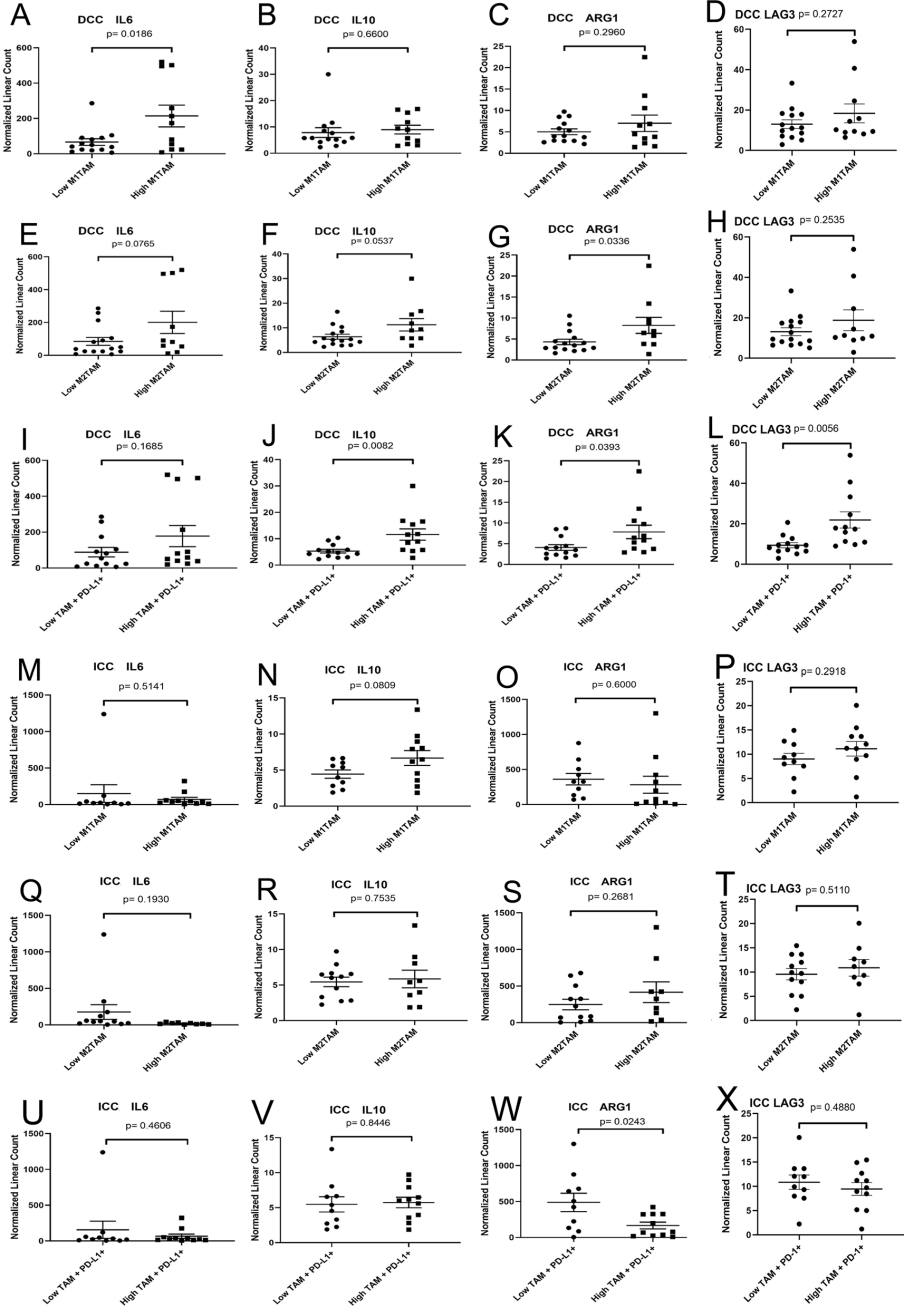
Comparison of selected gene expression between cholangiocarcinomas with higher versus lower density of TAMs and their subtypes. *t* tests were performed with *p* value indicated. DCC, distal cholangiocarcinoma; HC, hilar cholangiocarcinoma; ICC, intrahepatic cholangiocarcinoma

In order to examine the cytokine/chemokine and intracellular functional gene expression, we correlated the immune cell densities with mRNA expression levels in tumors analyzed by the Nanostring technologies. To this end, tissues from FFPE sections of the same DCC and ICC cases for multiplex IHC were subjected to Nanostring analysis. As shown in Fig. 7, we found the correlation between IL6, IL10 and ARG1 expression in tumors and TAMs. IL6 and IL10 are two pro-tumoral inflammatory cytokines that suppress effector T cell functions; and ARG1 is defining feature of immunosuppressive myeloid cells and leads to depletion of L-arginine, which is required for T cell and NK cell function and survival. In DCC, higher density of M2 TAM is associated with higher expression of IL6, IL10 and ARG1 (*p* = 0.0336). These results suggest that M2 TAMs, which are likely overlapped with PD-L1+ TAM, are associated with an increased T cell suppression mechanism similarly as the association of higher density of PD-L1+ TAM with higher expression of IL10, ARG1, and the T cell exhaustion factor LAG3 is related to poorer prognosis. By contrast, higher density of M1 TAMs, which is not prognostic as shown above, is also associated with higher expression of IL6, but not IL10 and ARG1. In ICC, the association of IL6, IL10, and ARG1 with TAMs appear to be different from that in DCC. On the contrary, in ICC, higher density of PD-L1^+^ TAMs is significantly associated with lower expression of ARG1 in ICC (*p* = 0.0245). This result may suggest that the major function of PD-L1+ TAMs is not mediated by ARG1, but mainly mediated by PD-L1. Consistent with this notion, the above multiplex IHC results showed that higher density of PD-L1^+^ M2 TAMs is significantly associated with lower density of non-exhausted CD8^+^PD-1^−^EOMES^−^ cells (Additional file 1: Fig. S16).

We thus further examined the intracellular functional gene expressions in ICC by conducting a multiple batch integration analysis of single cell RNA sequencing data from multiple studies [35, 36] that were previously published. We found that an increased expression of T cell exhaustion factors measured as the ratio of TOX2^+^ CD8^+^ cells to all CD8^+^ cells is associated significantly with a higher percentage of immature dendritic cells (DC) among all the single cells within each of the individual samples analyzed (Additional file 1: Fig. S17). The higher expression of other T cell exhaustion factors such as EOMES and LAG3 similarly measured are also in a strong trend associated with the higher percentage of immature DC. Moreover, the higher ratio of TIM3^+^ CD8^+^ cells to all CD8^+^ cells is associated significantly with the higher percentage of PD-L1^+^ M1 macrophages and associated in a strong trend with the higher percentage of PD-L1^+^ M2 macrophages. Interestingly, we found that there is a strong correlation between the RNA expression of EOMES and that of LAG3 in CD8^+^ cells in those tumor samples showing higher percentage of mature DCs, immature DC, PD-L1^+^ M1 macrophages or PD-L1^+^ M2 macrophages. There is also a strong correlation between the RNA expression of EOMES and that of TIM3 in CD8^+^ cells in those tumor samples showing a possibly more immunosuppressive TME including a higher percentage of immature DC, PD-L1^+^ M1 or M2 macrophages, but not in those samples showing a higher percentage of mature DC. These results suggest that EOMES, LAG3 and TIM3 are likely in the same T cell exhaustion pathway in ICC. Taken together, our Nanostring assay and re-analysis of RNA sequencing results suggest that, in ICC, PD-L1^+^ TAMs exert their immunosuppressive function likely through promoting T cell exhaustion.

## Discussion

To our knowledge, this study is the most comprehensive analysis of the tumor immune microenvironment of cholangiocarcinoma to date. By utilizing a broad array of myeloid and lymphoid biomarkers in multiplex IHC analysis within a large cholangiocarcinoma cohort, we present the atlas of the TME of cholangiocarcinoma. By employing multiplex IHC, multiple maker utilization allowed for a more precise definition of individual immune subtypes, associations among them, and their impacts on prognosis. Furthermore, one advantage of multiplex IHC is that it can minimize the inherent bias of quantification due to non-specific staining. By employing multiple biomarkers for individual cell identification, it becomes extremely unlikely that multiple antibodies have non-specific staining on the same cells. More importantly, multiplex IHC allows quantification of multiple immune cell subtypes within the same tissue area on the same tissue section and thus leads to a more precise correlative analysis between different immune subtypes. Although the analysis is limited to one tissue section, we have previously demonstrated that the areas chosen for quantification are highly representative of the whole tumor block [27]. The specimens analyzed in this study are all resected tumors from untreated patients. Although it should be noted that these specimens do not represent metastatic cholangiocarcinoma, the results from these specimens obtained from a relatively homogeneous patient population would more accurately demonstrate the role of TME in the patient’s clinical outcome. Limited by the tissue quantity and quality, biopsy specimens from metastatic cholangiocarcinoma would also be inadequate for a comprehensive TIME analysis. Nevertheless, the immune parameters analyzed in this study can be selectively validated in the biopsy specimens from metastatic cholangiocarcinoma.

Most importantly, this comprehensive multiplex IHC approach demonstrates many new findings which would otherwise be missed by traditional IHC approaches or small-panel multiplex IHC approaches. First, NK cells were found to occupy the largest immune cell compartment in all three types of cholangiocarcinoma. NK cells were poorly studied in cholangiocarcinoma. This study also does not demonstrate a prognostic value of the density of NK cells, suggesting NK cells naturally present in cholangiocarcinoma do not have a significant antitumor function. Therefore, future studies on how to re-engage NK cells should be considered. Second, this study suggests that TAM is a heterogeneous population and simply subgrouping into M1 and M2 macrophages or using PD-L1 expression to distinguish the prognostic values of TAM is not sufficient. One striking finding from this analysis was that PD-L1^−^ M2 macrophages are associated with a good prognosis in DCC and also in certain subgroups of ICC. Taking advantage of our multiplex IHC approach by using PD-L1+ M1 TAM, PD-L1+ M2 TAM, PD-L1- M1 TAM, PD-L1- M2 TAM, and/or Treg as biomarkers simultaneously to subgroup DCC and ICC, this study demonstrates the superiority and necessity of using multiple biomarkers to distinguish cholangiocarcinoma patients with different prognoses. Meanwhile, this study underscores the importance of targeting immunosuppressive TME components in the clinical trials of immunotherapy for cholangiocarcinoma, however, by using a combined biomarker strategy to select appropriate patient populations. Third, this study demonstrates that lower densities of immunosuppressive cells are associated with lower infiltration of effector T cells in DCC and HC, suggesting that targeting immunosuppressive cells without priming the TME with effector T cells in the immunotherapy for DCC and HC is not sufficient. As shown in this study, DCC and HC appear to have a “colder” TME than ICC, and thus would need to be treated with T cell priming agents such as vaccine therapy and T cell therapy. On another hand, this study suggests that targeting effector T cells or immune checkpoints alone is also unlikely sufficient because an increased infiltration of effector T cells is associated with increased infiltration of Tregs, and CSF-1R+ cells in DCC and HC. Taken together, the comprehensive analysis of TME in this study informs the design of clinical trials including a recently initiated clinical trial of testing durvalumab anti-PD-L1 antibody in combination with a CSF-1R inhibitor (SNDX-6532) following chemo or radio-embolization for patients with intrahepatic cholangiocarcinoma (NCT04301778).

This study is consistent with the majority of the previously published studies in that we showed that the infiltration of CD8^+^ T cells is a positive prognostic factor in cholangiocarcinoma and that PD-L1 is an indicator of poor prognosis [19, 22, 24]. However, previous studies only characterized CD8^+^ T cells in general; and simply characterizing CD8^+^ T cells in general is apparently not sufficient in predicting the prognosis of cholangiocarcinoma. In this study, we newly demonstrated that the density of CD8^+^Granzyme B^+^ T cells, which are considered to be high-quality effector T cells, to be a stronger prognostic indicator in cholangiocarcinoma. Non-exhausted CD8^+^PD-1^−^EOMES^−^ T cells were shown to be a stronger prognostic indicator in ICC. More importantly, this study demonstrated the importance of spatial analysis as only the density of CD8^+^PD-1^−^EOMES^−^ T cells in the tumor areas, but not in the peripheries of the tumors, is prognostic. In addition, this study validated some equivocal conclusions made in the previously published studies [37] including: the negative prognostic values of Tregs and the Th1/Th2 ratio in all types of cholangiocarcinoma, the negative prognostic value of CD4^+^PD-1^+^ cells in DCC, and the negative prognostic value of CD8^+^PD-1^+^ cells in DCC and ICC. The univariate and multivariate analyses of each immune cell parameter together with multiple clinicopathologic factors did not suggest that the correlation between any immune parameter tested and the survival was confounded by clinicopathologic factors or stages (Additional file 1: Tables S4–S33), indicating that the immune cell parameters mentioned above are independent prognostic factors.

This study is the first of its kind to compare the TME between various subtypes of cholangiocarcinoma, ICC, HC and DCC. A limitation of this study is that it does not include gall bladder cancer due to its rarity in the United States. However, comparison between the three major types of cholangiocarcinoma in this study has already revealed that different types of cholangiocarcinoma have distinct immune patterns within TME. Not only are the densities of individual immune cell subtypes different among different types of cholangiocarcinoma, but their prognostic implications are also different. This is not a surprise considering that ICC, HC, and DCC are known to originate from different cholangiocytes [38]. Therefore, different subtypes of cholangiocarcinomas shall not be combined together in clinical trials of immunotherapy without planned subgroup analyses. It is also conceivable that tumor genetics may influence on how tumor cells may reprogram the TME and warrant a future study through a large international consortium to further increase evaluable sample sizes.

There are several limitations with this study. A large part of the results presented here are exploratory in nature and require further investigation to draw concrete conclusions. Although the Nanostring analysis of mRNA expression of immune-related genes in FFPE tissues was incorporated into this study, a more comprehensive gene signature analysis with fresh tissues using the whole genomic transcriptomic approach is warranted. This would help interpreting the findings of this study and identifying new targets for immunotherapy. We also recognize the limitation of the cholangiocarcinoma cohort from a single US institution in investigating the association of different etiologies of cholangiocarcinoma with the immune status in the TME. Therefore, a followup study with tumors from a multi-institutional international study is warranted in the future by using the methodologies established in this current study. The combined immune biomarker analysis performed in this study was based on the past knowledges, and thus, has its limitation. We will use an unbiased approach or a deep learning and artificial intelligence classifier to identify optimized multiparameter immune biomarkers in the future study and validate them in a larger, multi-institutional study. This study also did not include patients who received neoadjuvant chemotherapy and/or radiation, which is rarely indicated for resectable cholangiocarcinoma. Only approximately 40% of patients received adjuvant chemotherapy, which varied in its type and length among patients due to lack of the standard of care guideline at the time when the patients in this study cohort underwent surgical resection; and therefore, adjuvant chemotherapy was not included as a clinical parameter in the multivariant analysis. Thus, it remains interesting to study the impact of chemotherapy and radiation on the prognostic values of immune compositions of cholangiocarcinoma.

In conclusion, this study has created a broad atlas of tumor infiltrating immune cells in cholangiocarcinoma, providing the largest known resource of its kind for the immunotherapy research community of cholangiocarcinoma. This study underscores the importance of targeting immunosuppressive TME components in the clinical trials of immunotherapy for cholangiocarcinoma by using a combined biomarker strategy to select appropriate patient populations. This study suggests the importance of targeting immunosuppressive cells in all types of cholangiocarcinoma and priming the TME with effector T cells as a combination immunotherapy strategy for distal cholangiocarcinoma and hilar cholangiocarcinoma.

## Supplementary Information

**Additional file 1**. Supplemental Figures and Tables.

## Data Availability

The multiplex immunohistochemistry imaging data are available to assess through a cloud service.
