# Supplementary material for: Immune cell atlas of cholangiocarcinomas reveals distinct tumor microenvironments and associated prognoses
Source: J Hematol Oncol. 2022 Mar 28;15:37. doi: 10.1186/s13045-022-01253-z (PMC8962046; doi:10.1186/s13045-022-01253-z)
Supplement: Supplementary file 1 — Additional file 1. Supplemental Figures and Tables. [file 13045_2022_1253_MOESM1_ESM.pdf]

Supplementary Figures

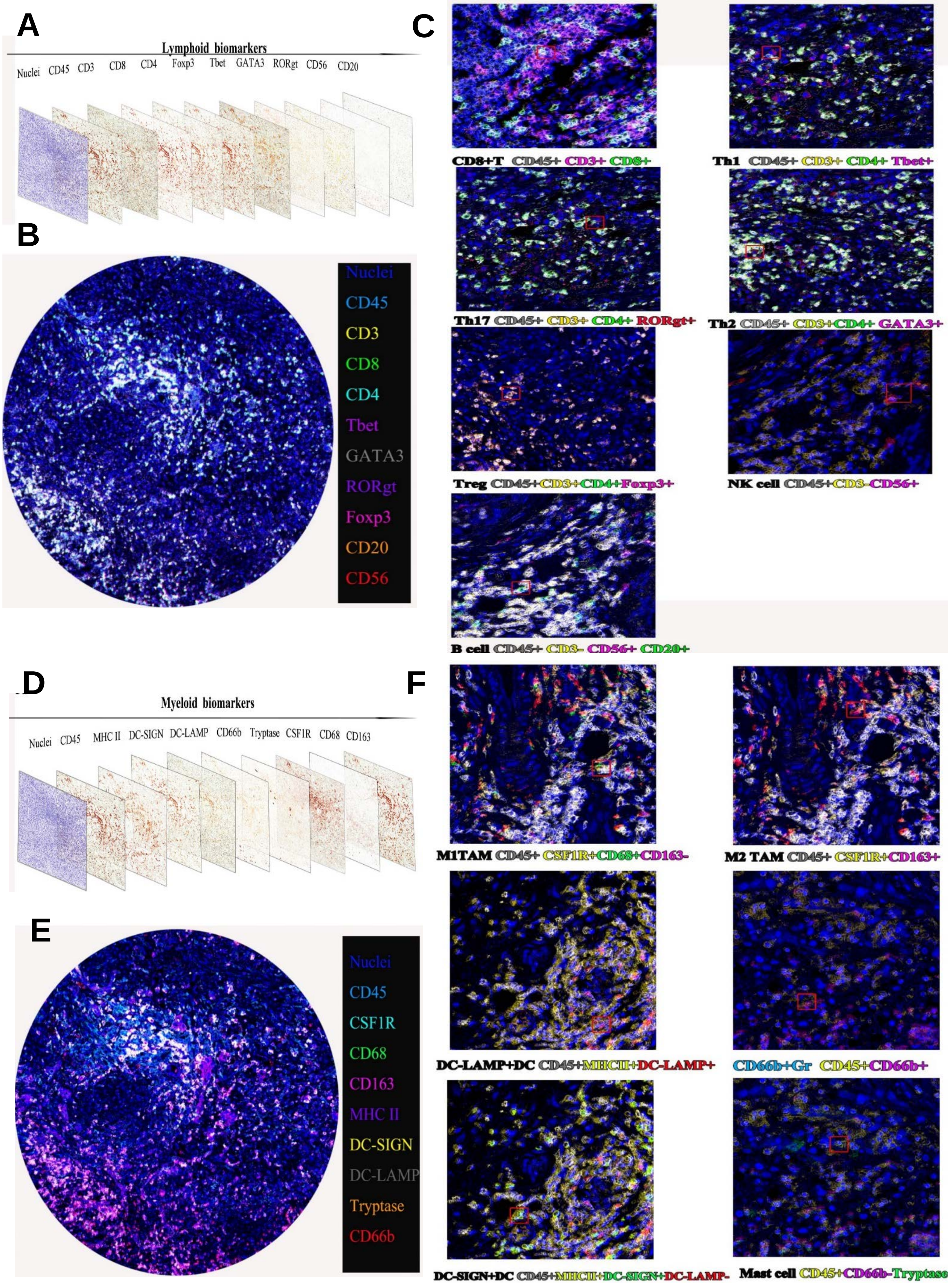

Figure S1 The working flow of multiplex immunohistochemistry staining with a lymphoid cell panel and a myeloid cell panel. A. Sequential staining and stripping of a series of immunohistochemistry staining with a panel of lymphoid cell markers. B. Overlaid multiple images stained with lymphoid cell markers with pseudocolors. C. Selected immune cell subtypes defined by multiple lymphoid cell markers. D. Sequential staining and stripping of a series of immunohistochemistry staining with a panel of myeloid cell markers. E. Overlaid multiple images stained with myeloid cell markers with pseudocolors. F. Selected immune cell subtypes defined by multiple myeloid cell markers.

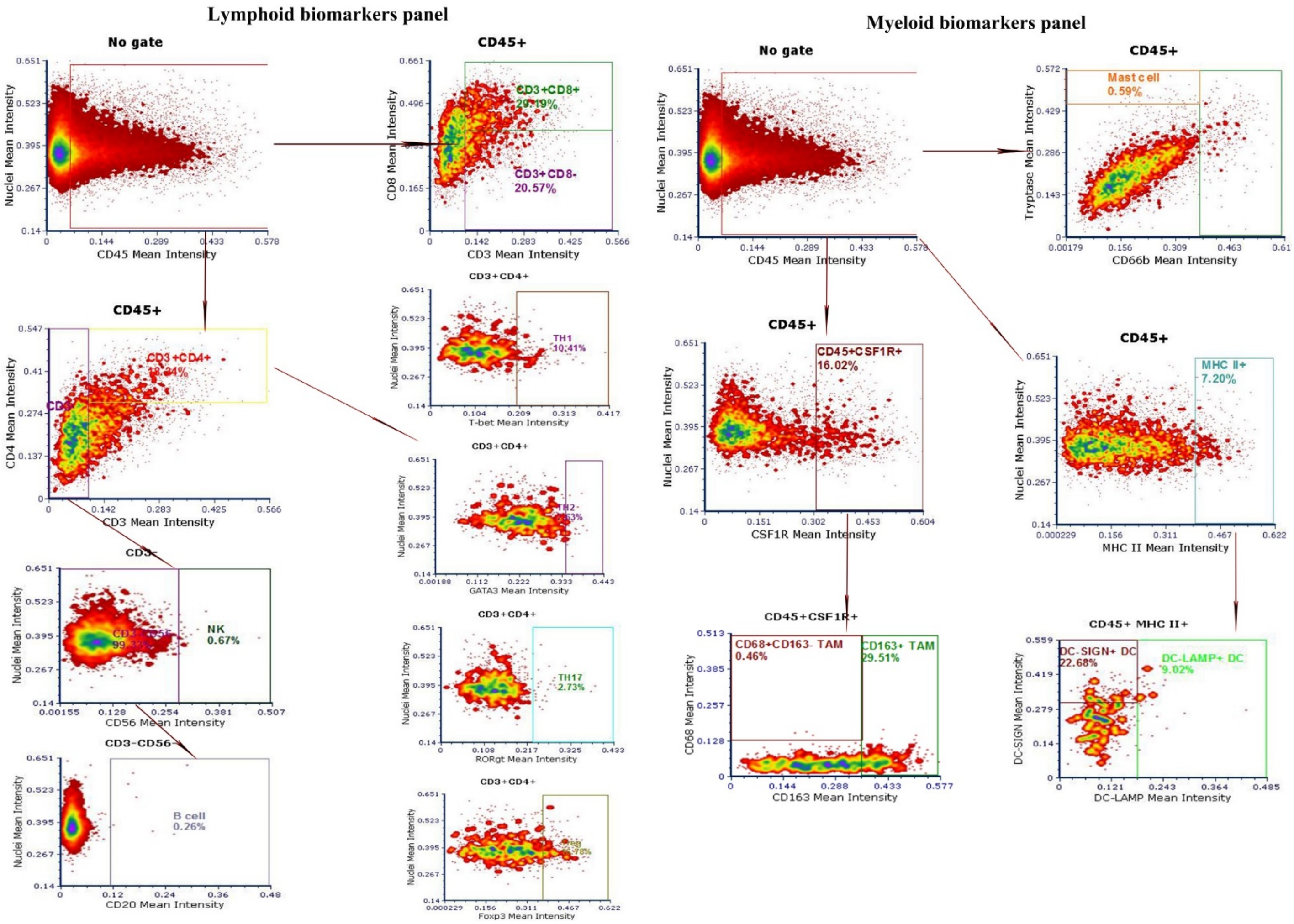

Figure S2 Representative gating information in the imaging cytometry analysis of quantified multiplex immunohistochemistry staining signals.

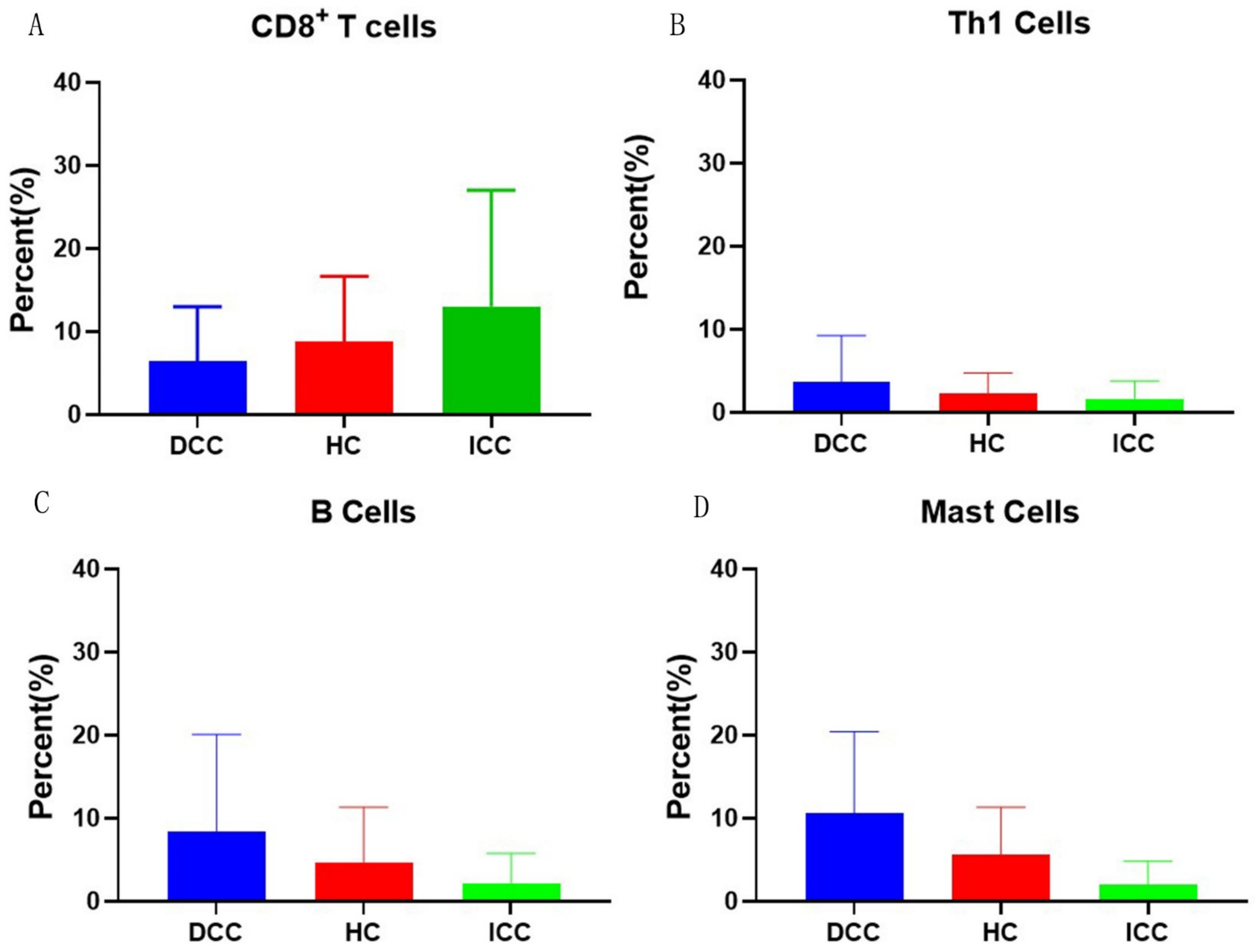

Figure S3 Percentages of immune cell subtypes among CD45+ cells. Statistically significant difference in the density of CD8+ cells, Th1 cells, B cells, and master cells comparing between any two of three cholangiocarcinoma subtypes (all $p<0.05$). DCC, distal cholangiocarcinoma; HC, hilar cholangiocarcinoma; ICC, intrahepatic cholangiocarcinoma.

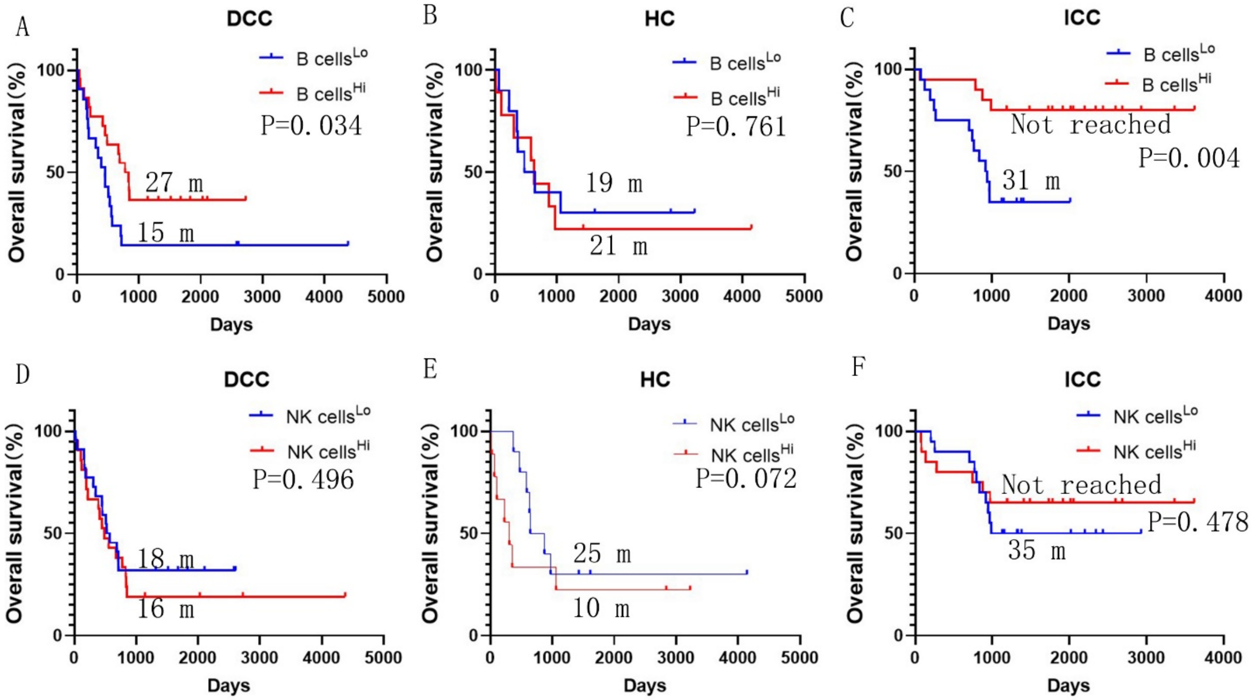

Figure S4 Kaplan-Meier curves of overall survival of patients whose tumors are grouped by higher density vs. lower density of B cells and NK cells as indicated. DCC, distal cholangiocarcinoma; HC, hilar cholangiocarcinoma; ICC, intrahepatic cholangiocarcinoma.

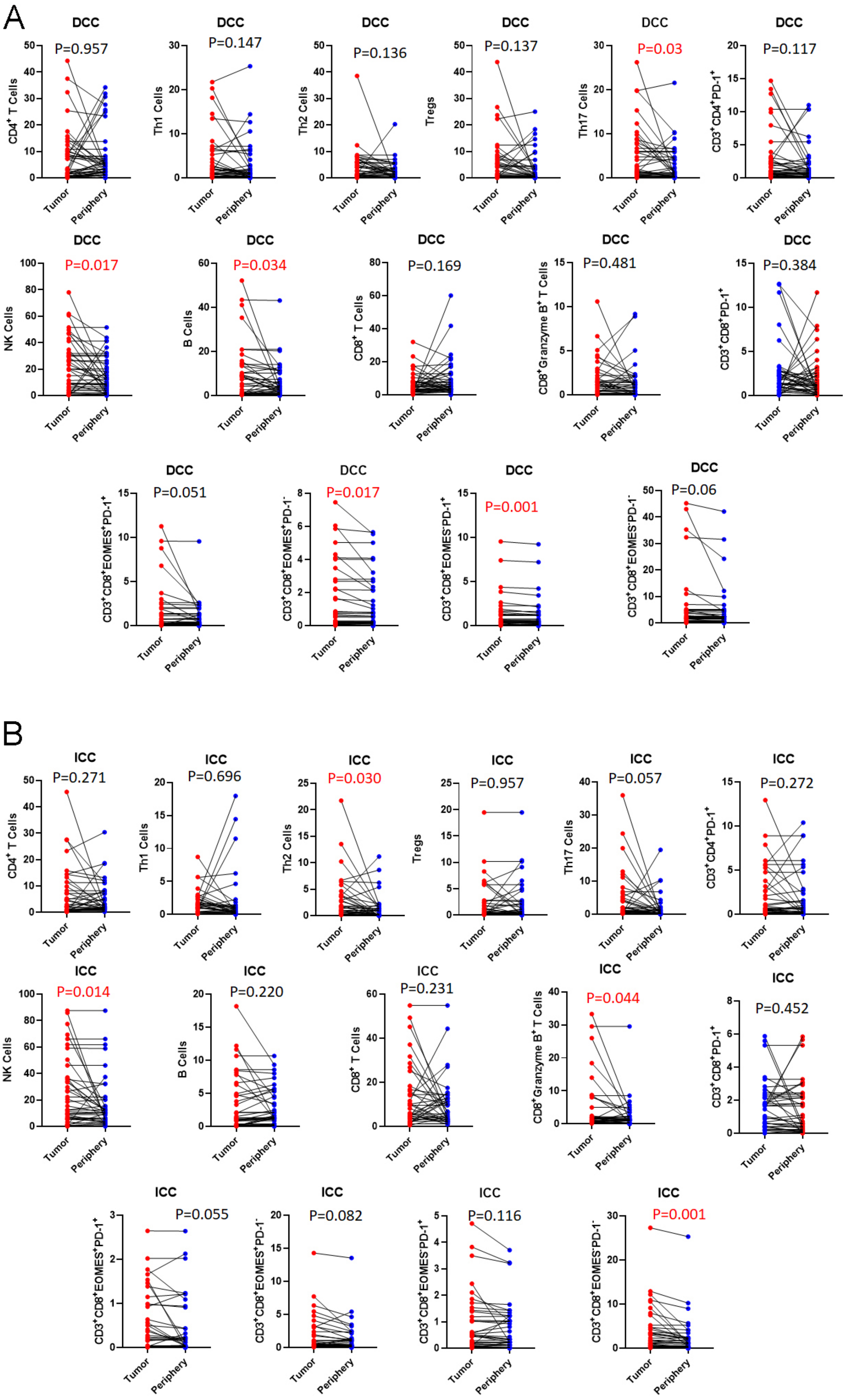
A
DCC
P=0.957
CD4+ T Cells
DCC
P=0.147
Th1 Cells
DCC
P=0.136
Th2 Cells
DCC
P=0.137
Tregs
DCC
P=0.03
Th17 Cells
DCC
P=0.117
CD3+CD4+PD-1+
DCC
P=0.017
NK Cells
DCC
P=0.034
B Cells
DCC
P=0.169
CD8+ T Cells
DCC
P=0.481
CD8+Granzyme B+ T Cells
DCC
P=0.384
CD3+CD8+PD-1+
DCC
P=0.051
CD3+CD8+EOMES+PD-1+
DCC
P=0.017
CD3+CD8+EOMES+PD-1-
DCC
P=0.001
CD3+CD8+EOMES-PD-1+
DCC
P=0.06
CD3+CD8+EOMES-PD-1-
B
ICC
P=0.271
CD4+ T Cells
ICC
P=0.696
Th1 Cells
ICC
P=0.030
Th2 Cells
ICC
P=0.957
Tregs
ICC
P=0.057
Th17 Cells
ICC
P=0.272
CD3+CD4+PD-1+
ICC
P=0.014
NK Cells
ICC
P=0.220
B Cells
ICC
P=0.231
CD8+ T Cells
ICC
P=0.044
CD8+Granzyme B+ T Cells
ICC
P=0.452
CD3+CD8+PD-1+
ICC
P=0.055
CD3+CD8+EOMES+PD-1+
ICC
P=0.082
CD3+CD8+EOMES+PD-1-
ICC
P=0.116
CD3+CD8+EOMES-PD-1+
ICC
P=0.001
CD3+CD8+EOMES-PD-1-
Tumor
Periphery

Figure S5 Comparison of lymphocyte infiltrations in the tumor area vs. the periphery. The tumor areas and the peripheries of tumors circled under pathologist guidance. The densities of various T cell subtypes, B cells, and NK cells as indicated were compared between tumor areas and peripheries of tumors. Paired t tests were done. Tumor areas and peripheries on the same cases were connected by a line. **A.** DCC, distal cholangiocarcinoma; **B.** ICC, intrahepatic cholangiocarcinoma.

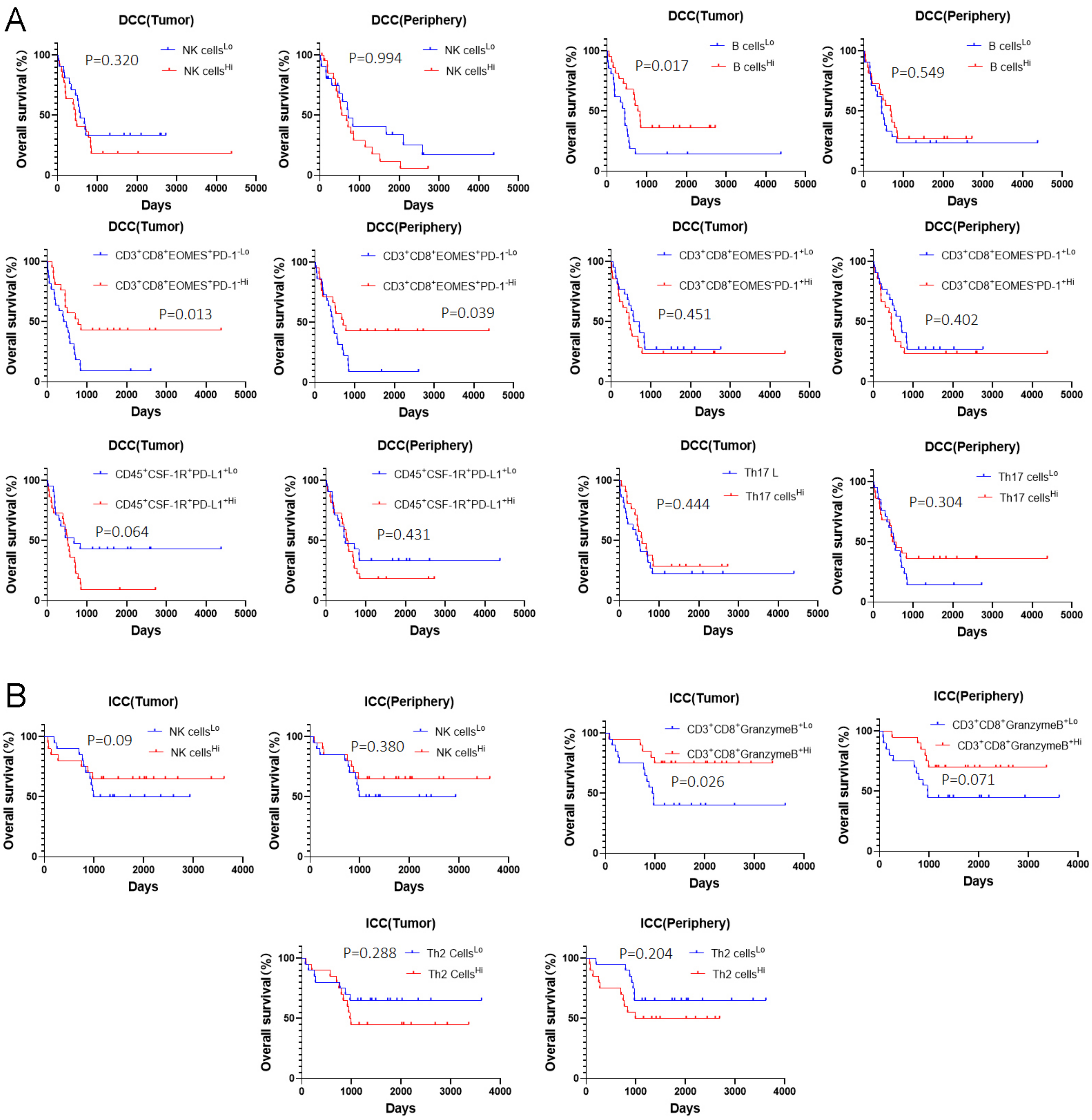

Figure S6 Kaplan-Meier curves of overall survival of patients whose tumors are grouped by higher density vs. lower density of various T cell subtypes, B cells and NK cells as indicated in the tumor areas and in the peripheries of the tumors, respectively. The survival correlation of those immune cell subtypes that showed significant difference in the filtration between the tumor areas and the peripheries of the tumors was analyzed here. **A.** DCC, distal cholangiocarcinoma; **B.** ICC, intrahepatic cholangiocarcinoma.

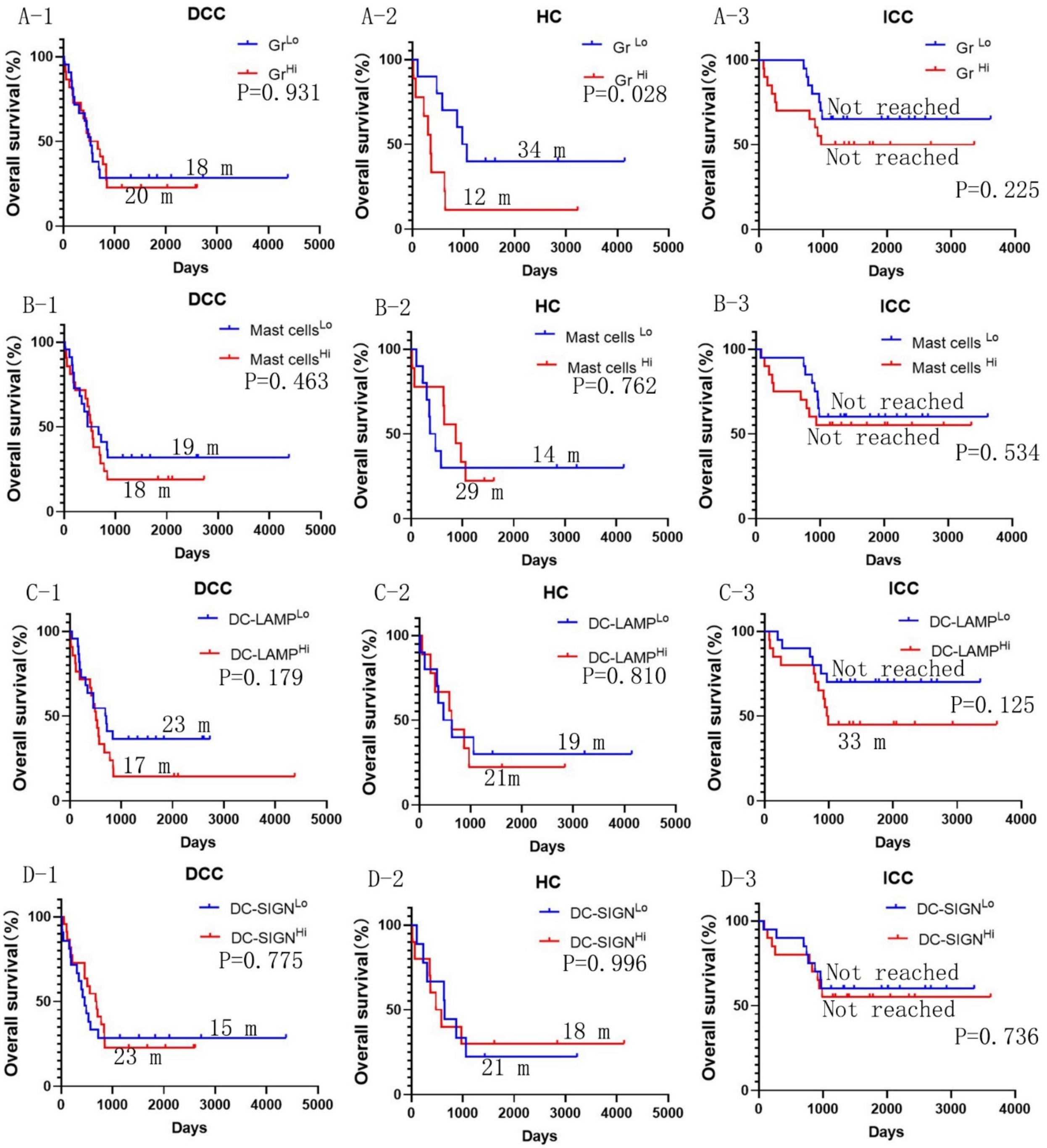

Figure S7 Kaplan-Meier curves of overall survival of patients whose tumors are grouped by higher density vs. lower density of myeloid cell subtypes as indicated. Log rank tests were done with p values indicated. DCC, distal cholangiocarcinoma; HC, hilar cholangiocarcinoma; ICC, intrahepatic cholangiocarcinoma.

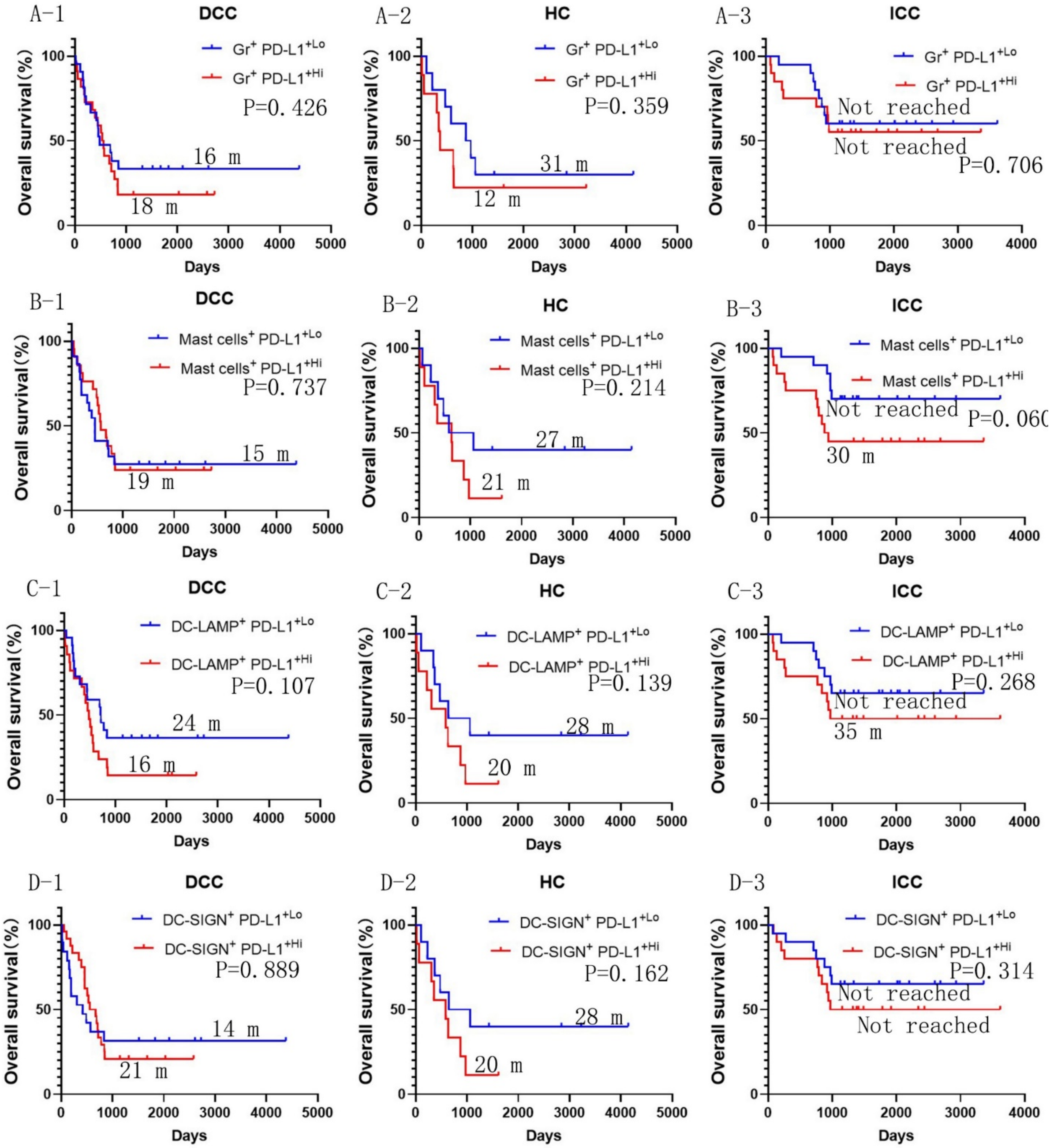

Figure S8 Kaplan-Meier curves of overall survival of patients whose tumors are grouped by higher density vs. lower density of PD-L1+ myeloid cell subtypes as indicated. Log rank tests were done with p values indicated. DCC, distal cholangiocarcinoma; HC, hilar cholangiocarcinoma; ICC, intrahepatic cholangiocarcinoma.

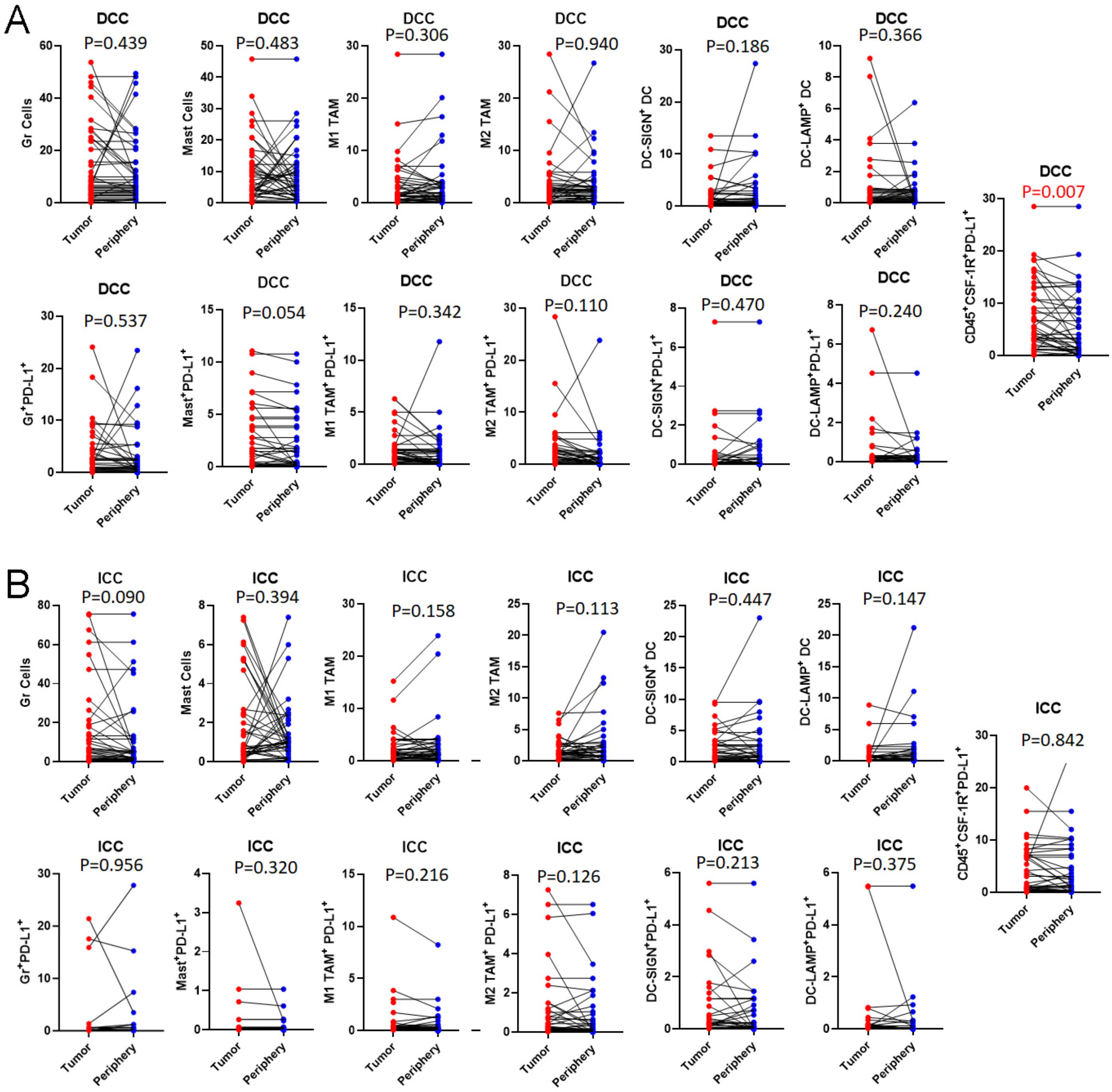

Figure S9 Comparison of myeloid cell infiltrations in the tumor area vs. the periphery. The tumor areas and the peripheries of tumors circled under pathologist guidance. The densities of various myeloid cell subtypes as indicated were compared between tumor areas and peripheries of tumors. Paired t tests were done. Tumor areas and peripheries on the same cases were connected by a line. **A.** DCC, distal cholangiocarcinoma; **B.** ICC, intrahepatic cholangiocarcinoma.

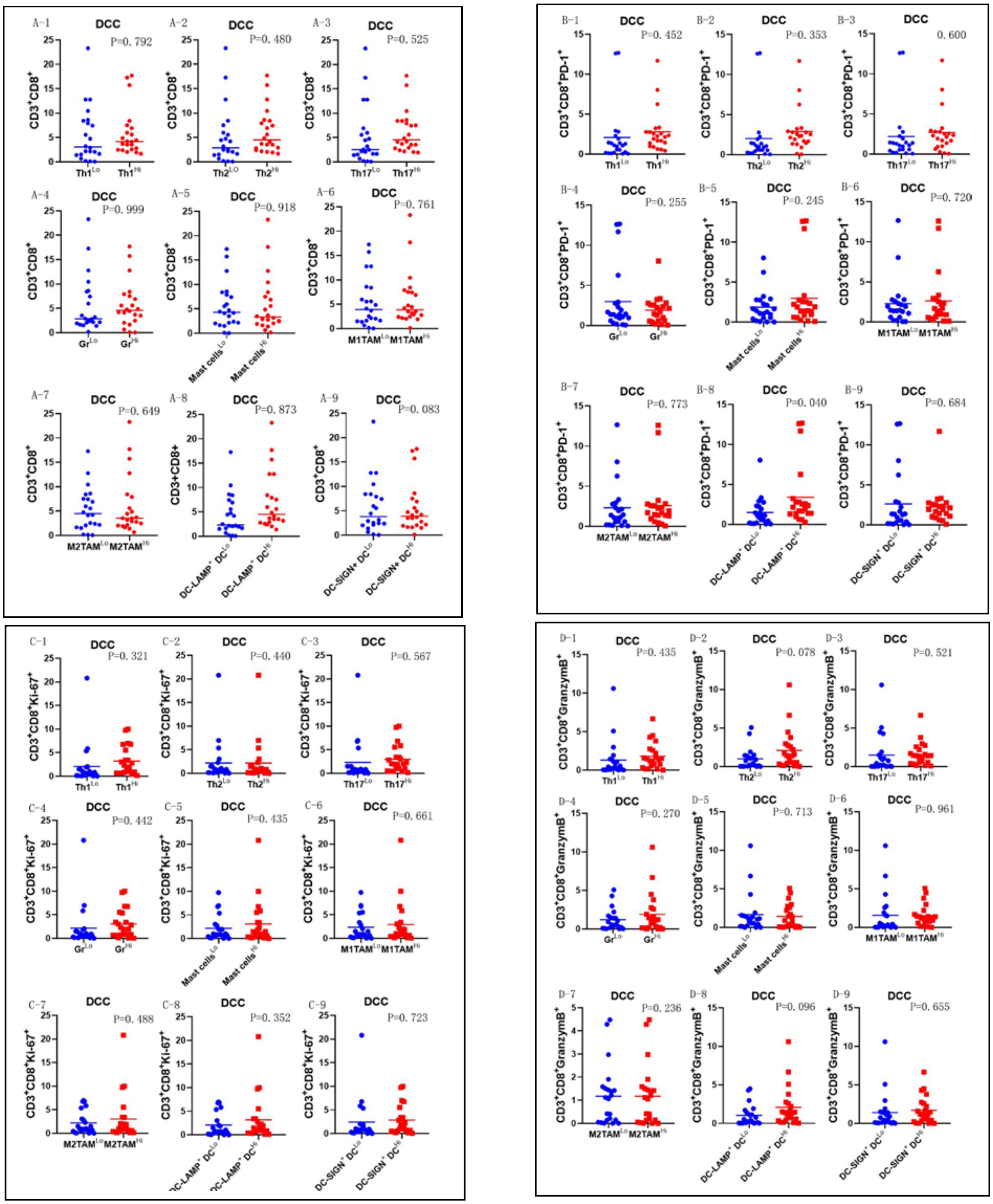

Figure S10 Comparing various effector T cell subtypes between distal cholangiocarcinomas (DCC) with higher vs. lower density of regulatory immune cells. t tests were performed with p value indicated.

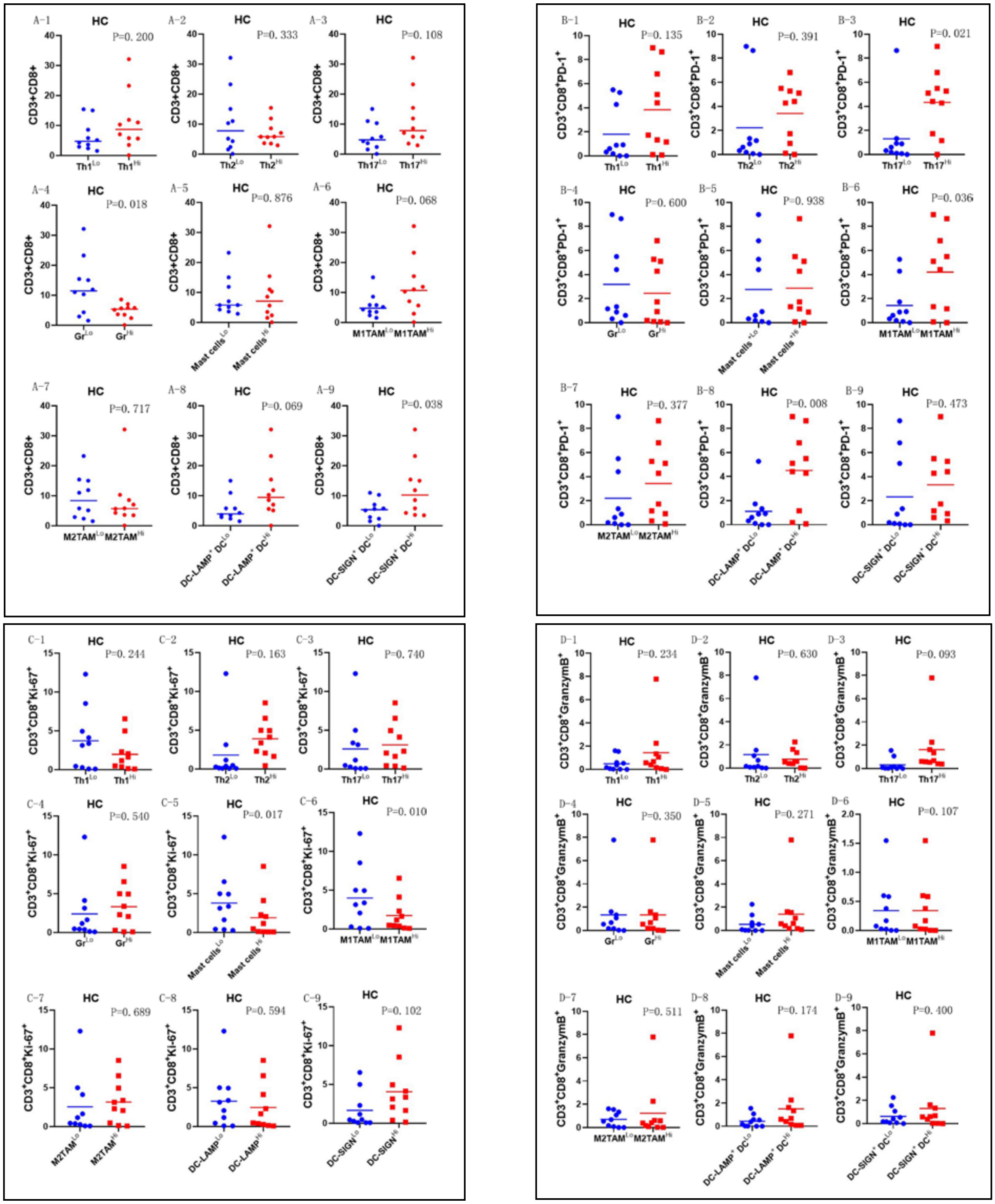

Figure S11 Comparing various effector T cell subtypes between hilar cholangiocarcinomas (HC) with higher vs. lower density of regulatory immune cells. t tests were performed with p value indicated.

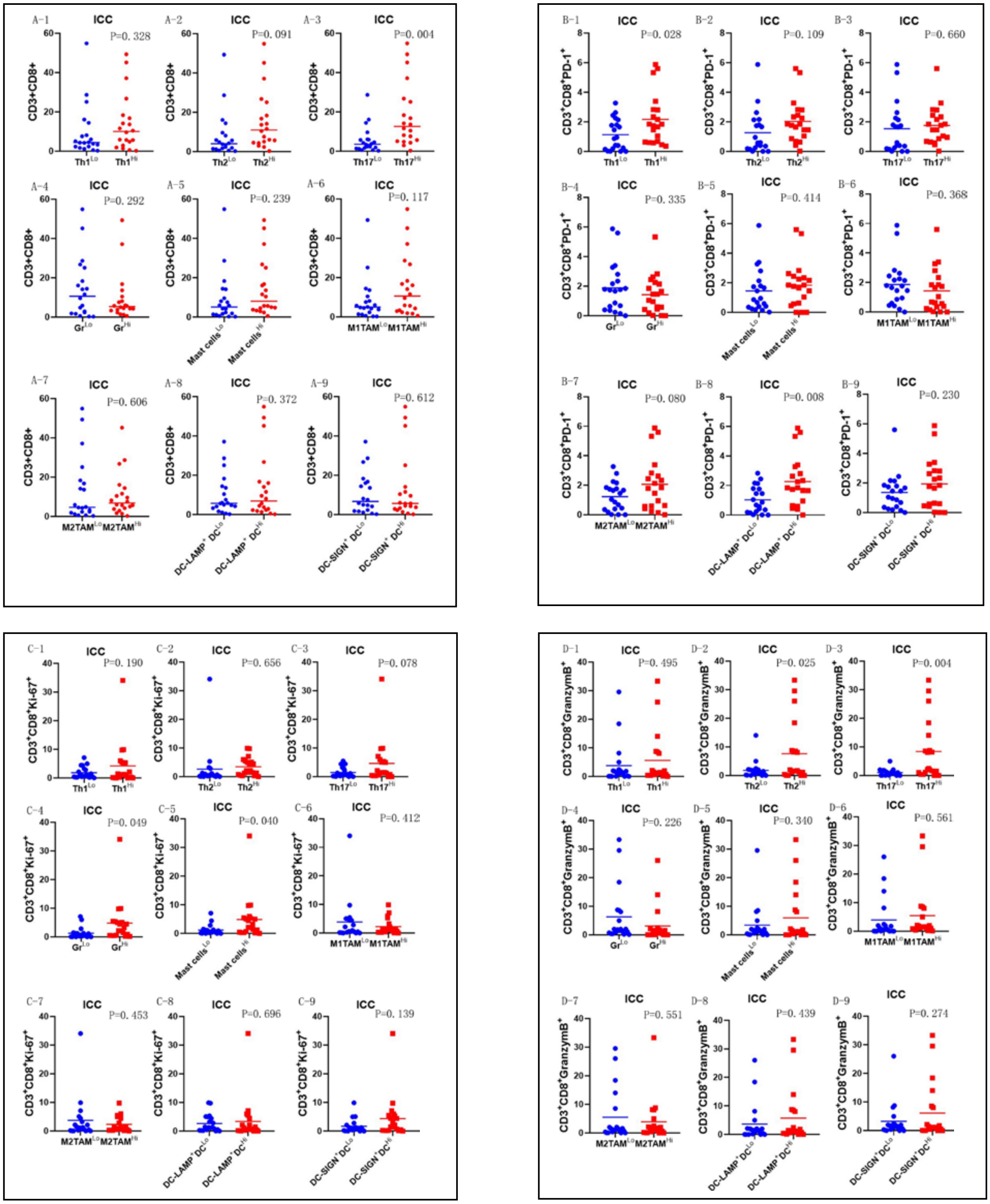

Figure S12 Comparing various effector T cell subtypes between intrahepatic cholangiocarcinomas (ICC) with higher vs. lower density of regulatory immune cells. t tests were performed with p value indicated.

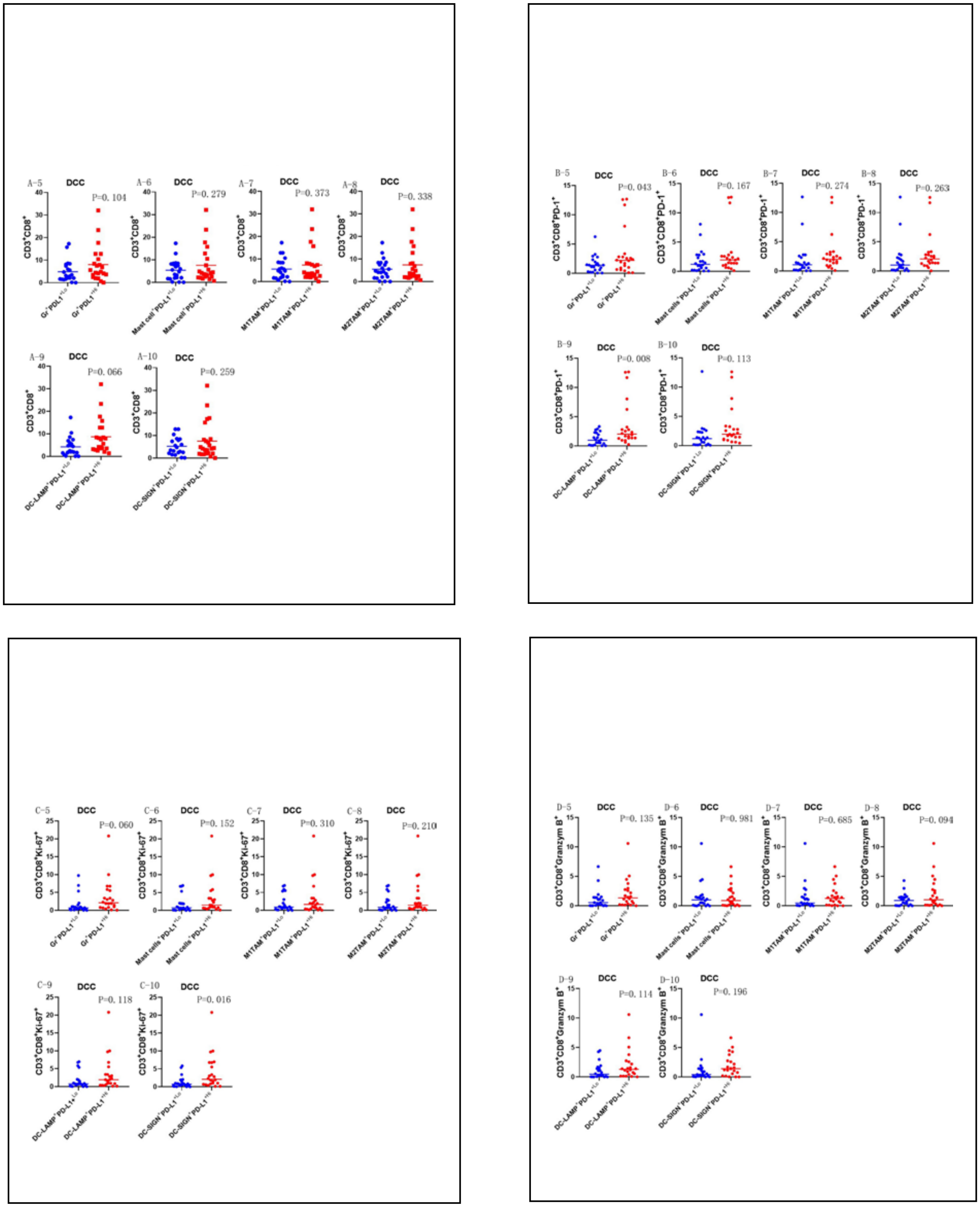

Figure S13 Comparing various effector T cell subtypes between distal cholangiocarcinomas (DCC) with higher vs. lower density of regulatory immune cells positive for PD-L1. t tests were performed with p value indicated.

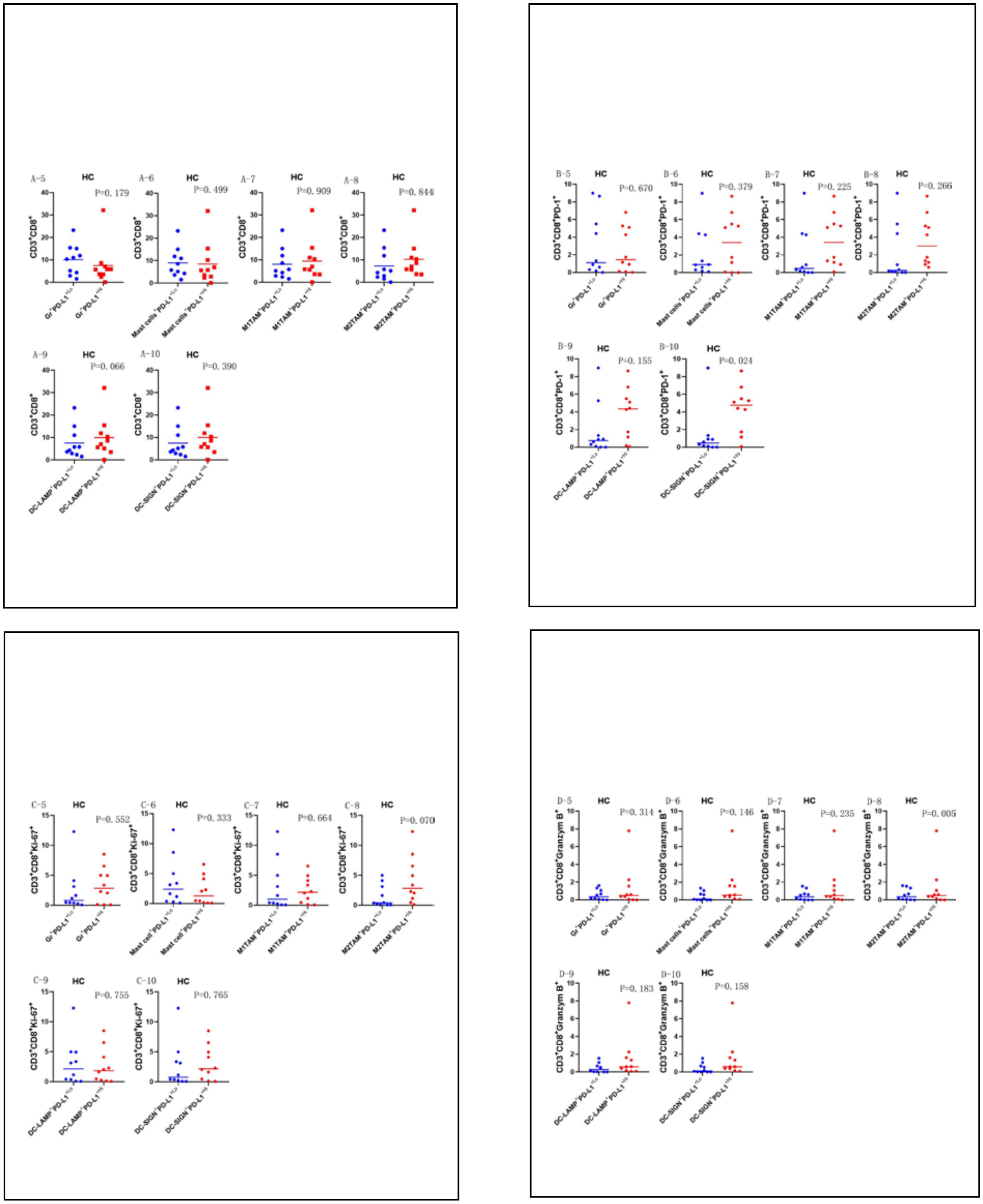

Figure S14 Comparing various effector T cell subtypes between hilar cholangiocarcinomas (HC) with higher vs. lower density of regulatory immune cells positive for PD-L1. t tests were performed with p value indicated.

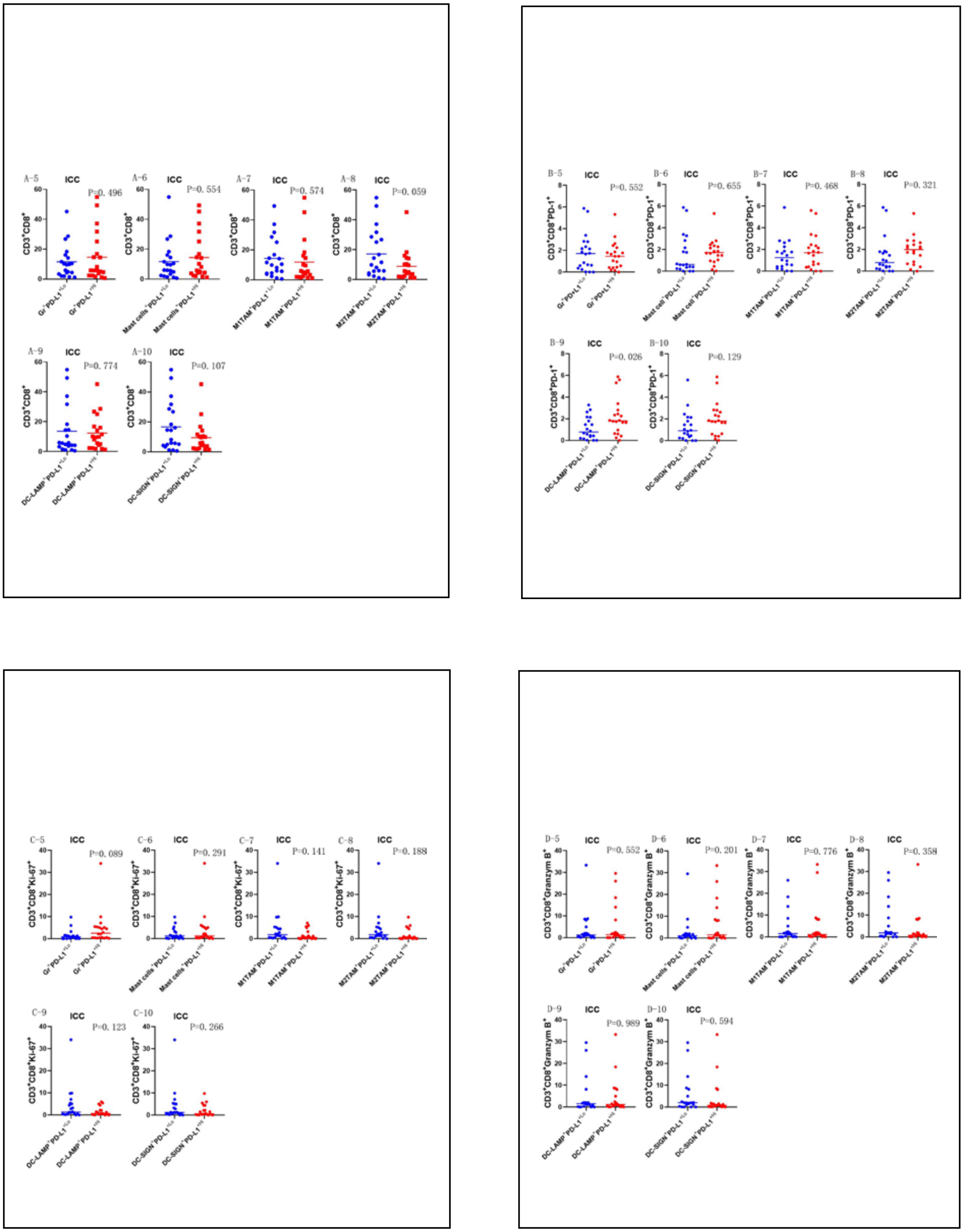

Figure S15 Comparing various effector T cell subtypes between intrahepatic cholangiocarcinomas (ICC) with higher vs. lower density of regulatory immune cells positive for PD-L1. t tests were performed with p value indicated.

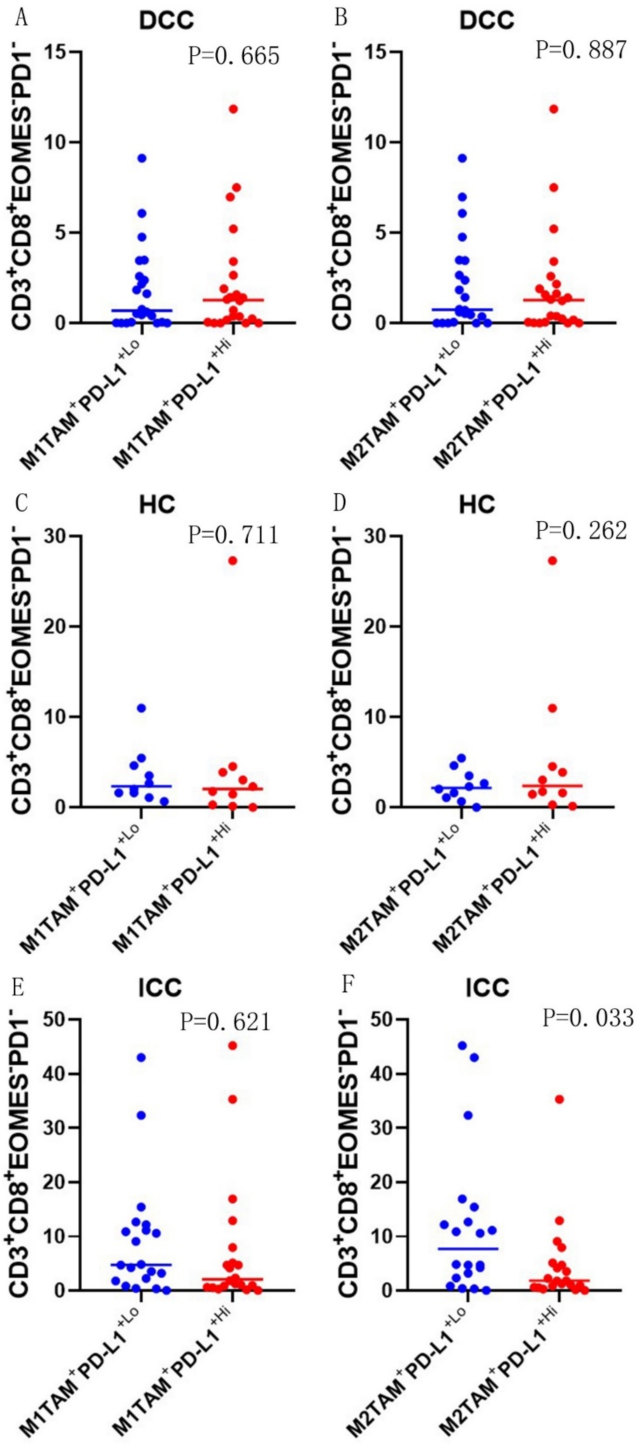

Figure S16 Comparing non-exhausted CD8+ cells between cholangiocarcinoma with higher vs. lower density of TAMs positive for PD-L1. t tests were performed with p value indicated. DCC, distal cholangiocarcinoma; HC, hilar cholangiocarcinoma; ICC, intrahepatic cholangiocarcinoma.

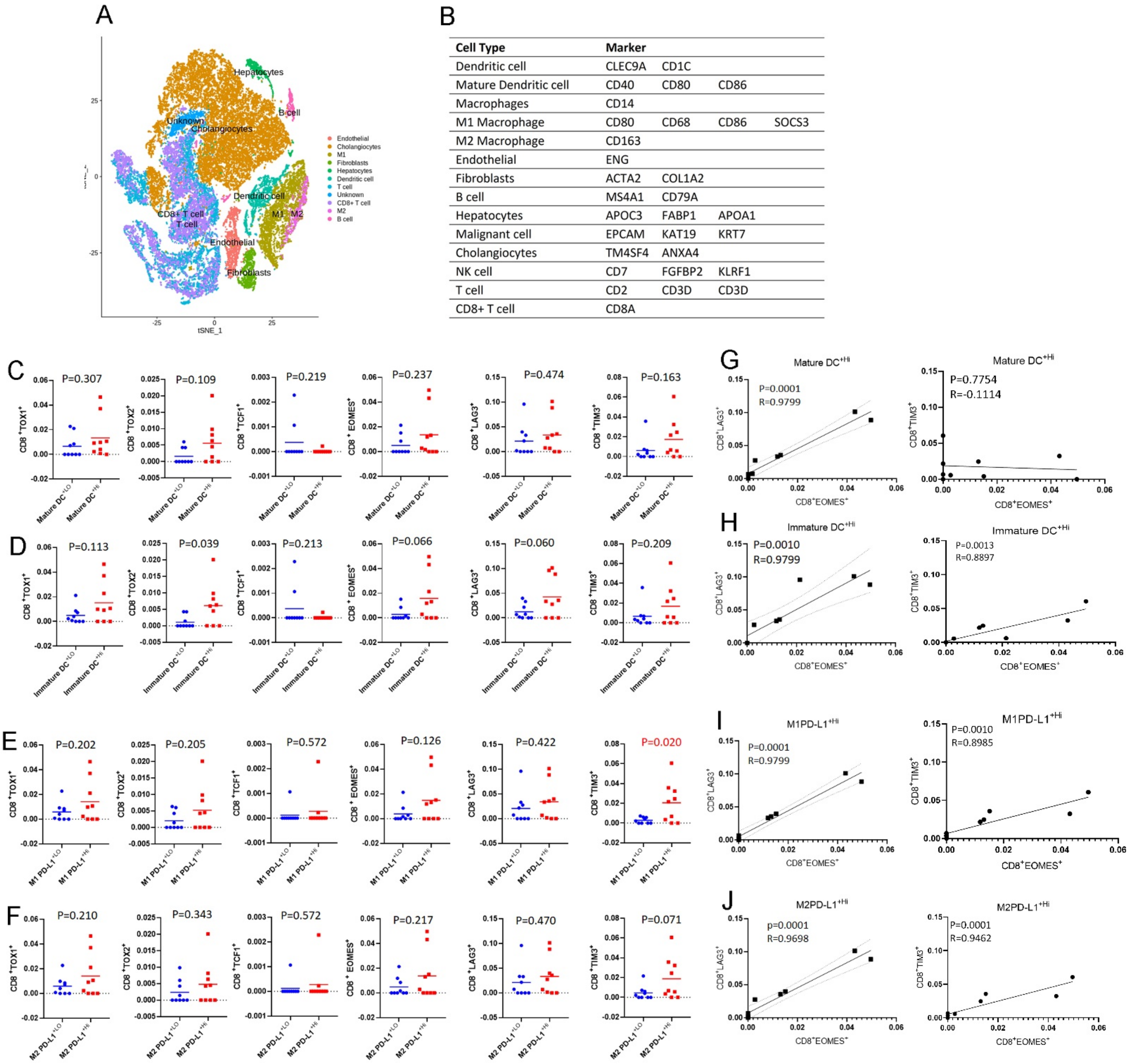

Figure S17 Single cell RNA sequencing analysis of T cell exhaustion signals in CD8+ cells and their correlations with the density of dendritic cells and TAMs in intrahepatic cholangiocarcinoma. Single cell RNA sequencing results of human intrahepatic cholangiocarcinoma (ICC) were obtained from GSE138709[1] (n=10) and GSE125449[2] (n=10) and combined. The Seurat R package (version 4.0.1) was used for batch integration and the dimensionality reduction analysis. Those single cells that had the transcripts of > 200 genes were filtered in and a total of 40024 cells were analyzed. **A.** Cells were clustered by the t-Distributed Stochastic Neighbor Embedding (t-SNE) method. **B.** Markers that were used to identify each cell type. Selection of these markers was made according to the literature[1]. **C.** The 18 ICC samples were divided into two groups, those with a higher percentage of mature dendritic cells (DC) (Mature DC^{Hi}) and those with a lower percentage of mature dendritic cells (Mature DC^{Lo}) among all cells in the respective samples, by using the median percentage of mature DCs as the cutoff. **D.** The 18 ICC samples were divided into two groups, those with a higher percentage of immature dendritic cells (DC) (Immature DC^{Hi}) and those with a lower percentage of immature dendritic cells

(Immature DC^{Lo}) among all cells in the respective samples, by using the median percentage of inmature DCs as the cutoff. **E.** The 18 ICC samples were divided into two groups, those with a higher percentage of PD-L1+ M1 macrophages (M1 $PD\text{-}L1+^{Hi}$) and those with a lower percentage of PD-L1+ M1 macrophages (M1 $PD\text{-}L1+^{Lo}$) among all cells in the respective samples, by using the median percentage of PD-L1+ M1 macrophages as the cutoff. **F.** The 18 ICC samples were divided into two groups, those with a higher percentage of PD-L1+ M2 macrophages (M2 $PD\text{-}L1+^{Hi}$) and those with a lower percentage of PD-L1+ M2 macrophages (M2 $PD\text{-}L1+^{Lo}$) among all cells in the respective samples, by using the median percentage of PD-L1+ M2 macrophages as the cutoff. In **C, D, E, F**, the ratios of the number of CD8+ T cells that express the T cell exhaustion signals as indicated to the total number of CD8+ T cells within each sample were compared between the two groups described above by unpaired t tests. The correlation between the ratios of the number of CD8+ T cells that express EOMES to the total number of CD8+ T cells and the ratios of the number of CD8+ T cells that express LAG3 or TIM3 as indicated to the total number of CD8+ T cells within each sample in the groups with **(G)** Mature DC^{Hi}, **(H)** immature DC^{Hi}, **(I)** M1 $PD\text{-}L1+^{Hi}$, or **(J)** M2 $PD\text{-}L1+^{Hi}$, respectively, was assessed by linear regression. p values <0.01 and co-efficient R values close to 1.0 suggest a strong correlation between EOMES and LAG3 in all four groups and between EOMES and TIM3 in all the groups except Mature DC^{Hi}.

References in Supplementary Materials

1. Zhang, M., *et al.* Single-cell transcriptomic architecture and intercellular crosstalk of human intrahepatic cholangiocarcinoma. *J Hepatol* **73**, 1118-1130 (2020).
2. Ma, L., *et al.* Tumor Cell Biodiversity Drives Microenvironmental Reprogramming in Liver Cancer. *Cancer Cell* **36**, 418-430 e416 (2019).

Supplementary Table S1 Patient Clinicopathologic Characteristics

Characteristics	Distal Cholangiocarcinoma (DCC)（N=44）	Hilar Cholangiocarcinoma (HC)（N=20）	Intrahepatic Cholangiocarcinoma (ICC)（N=40）
**Gender**			
**Male**	28	13	17
**Female**	16	7	23
**Age(years)**	66.50	66.20	62.68
**TNM Stage**			
**I**	0	2	20
**II**	35	9	12
**III**	9	9	8
**Resection margin**			
**R0**	36	9	30
**R1**	7	11	10
**R2**	1	0	0
**Tumor sizes(cm)**	2.2	2.9	4.8
**Histologic grade**			
**Well**	5	5	5
**Moderately**	23	10	24
**Poor**	16	5	11
**Adjuvant chemo**			
**Yes**	20	8	14
**No**	22	12	26

Supplementary Table S2 Multiplex Immunohistochemistry Procedures and Antibody Information

Table S2-1 The Multiplex IHC Master Panel – One single panel including both lymphoid and myeloid cell markers

	**Cycle 0**	**Cycle1**	**Cycle2**	**Cycle3**	**Cycle4**
Primary antibody	Hematoxylin	CD68	PD-1	PD-L1	CD163
Clone/Product#	S3301	PG-M1	NAT105	E1l3N	10D6
Vendor	Dako	Abcam	Abcam	Cell Signaling	Thermo Scientific
Concentration(mg/ml)		0.000266	0.02	0.00874	1/100*
Primary Ab Reaction Time	1 min	30 min	2 h	2 h	30 min
HISTOFINE Secondary Ab and Reaction Time		Anti-mouse 30 min	Anti-mouse 30 min	Anti-rabbit 30 min	Anti-mouse 30 min
AEC reaction time		20 min	1 h	1 h	30 min

	**Cycle5**	**Cycle6**	**Cycle7**	**Cycle8**	**Cycle9**
Primary antibody	DC-LAMP	DC-SIGN	Tbet	MHCII	Foxp3
Clone/Product#	1010e1.01	DC-28	4B/10	ERP112266	236A/E7
Vendor	Novus Biological	Santa Cruz	Santa Cruz	Abcam	eBioscience
Concentration(mg/ml)	0.005	0.002	0.002	0.00026	0.0125
Primary Ab Reaction Time	30 min	30 min	30 min	30 min	30 min
HISTOFINE Secondary Ab and Reaction Time	Anti-rat 30 min	Anti-mouse 30 min	Anti-mouse 30 min	Anti-rabbit 30 min	Anti-mouse 30 min
AEC reaction time	30 min	1 h	30 min	20 min	20 min

	**Cycle10**	**Cycle11**	**Cycle12**	**Cycle 13**	**Cycle14**
Primary antibody	CD4	CD8	Granzyme B	CSF1R	CD3
Clone/Product#	4B12	C8/144b	EP230	SP211	Sp7
Vendor	Thermo Scientific	Thermo Scientific	Sigma Aldrich	Abcam	Thermo Scientific
Concentration(mg/ml)	1/25*	0.005	0.004552	0.00064	1/150*

Primary Ab Reaction Time	30min	30min	30min	30min	30min
HISTOFINE Secondary Ab and Reaction Time	Anti-mouse 30min	Anti-mouse 30min	Anti-mouse 30min	Anti-rabbit 30min	Anti-rabbit 30min
AEC reaction time	30 min	30 min	30 min	20 min	40 min

	Cycle15	Cycle16	Cycle17	Cycle18	Cycle19
Primary antibody	GATA3	CD66b	CD56	CD20	Tryptase
Clone/Product#	L50-823	G10f5	123c3	0.N.85	AA1
Vendor	BD	eBioscience	Santa Cruz	Santa Cruz	Abcam
Concentration(mg/ml)	0.005	0.016667	0.004	0.0005	0.00005
Primary Ab Reaction Time	30 min	30 min	30 min	30 min	30 min
HISTOFINE Secondary Ab and Reaction Time	Anti-mouse 30 min	Anti-mouse 30 min	Anti-mouse 30 min	Anti-mouse 30 min	Anti-mouse 30 min
AEC reaction time	30 min	20 min	20 min	1 h	20 min

	Cycle20	Cycle21	Cycle22
Primary antibody	RORgt	Ki67	CD45
Clone/Product#	6F3.1	Sp6	H130
Vendor	EMD Millipore	Abcam	Thermo Scientific
Concentration(mg/ml)	0.0005	0.0003	0.005
Primary Ab Reaction Time	30 min	1 h	30 min
HISTOFINE Secondary Ab and Reaction Time	Anti-mouse 30 min	Anti-mouse 30 min	Anti-mouse 30 min
AEC reaction time	20 min	30 min	30 min

Table S2-2 The Multiplex IHC Panel I – The lymphoid cell marker panel

	Cycle1	Cycle2	Cycle3	Cycle4	Cycle5
Primary antibody	CD68	PD-L1	Granzyme B	Gata3	CD66b
Clone/Product#	PG-M1	E1l3N	EP230	L50-823	G10f5
Vendor	Abcam	Abcam	Cell signaling	Invitrogen	Dendritics
Concentration(mg/ml)	0.000266	0.00874	0.004552	0.005	0.016667
Primary Ab Reaction Time	30 min	2 h	1 h	30 min	30 min

Secondary Ab Reaction Time	30 min	30 min	30 min	30 min	30 min
AEC reaction time	45 min	2 h	2 h	90 min	30 min

	Cycle6	Cycle7	Cycle8	Cycle9	Cycle10
Primary antibody	CD3	CD56	CD20	Ki-67	FOXP3
Clone/Product#	Sp7	123c3	0.N.85	Sp6	236A/E7
Vendor	Thermo Scientific	Invitrogen	Santa Cruz	Abcam	eBioscience
Concentration(mg/ml)	1/150*	0.004	0.0005	0.0003	0.0125
Primary Ab Reaction Time	30 min	30 min	30 min	1 h	30 min
HISTOFINE Secondary Ab and Reaction Time	30 min	30 min	30 min	30 min	30 min
AEC reaction time	2 h	25 min	1 h	30 min	2 h

	Cycle11	Cycle12	Cycle13	Cycle 14	Cycle15
Primary antibody	CD4	Tryptase	CD8	Rorgt	EpCAM
Clone/Product#	4B12	AA1	C8/144b	6F3.1	E144
Vendor	Invitrogen	Abcam	eBioscience	EMD	Abcam
Concentration(mg/ml)	1/25*	0.00005	0.005	0.0005	0.000198
Primary Ab Reaction Time	30 min	30 min	30 min	30 min	60 min
HISTOFINE Secondary Ab and Reaction Time	30 min	30 min	30 min	30 min	30 min
AEC reaction time	60 min	20 min	45 min	20 min	25 min

Table S2-3 The Multiplex IHC Panel II – The myeloid cell marker panel

	Cycle1	Cycle2	Cycle3	Cycle4	Cycle5
Primary antibody	CD68	PD1	PDL1	CD163	DC-LAMP
Clone/Product#	PG-M1	NAT105	E1l3N	10D6	1010e1.01
Vendor	Abcam	Abcam	Cell Signaling	Thermo	Dendritics
Concentration(mg/ml)	0.000266	0.02	0.00874	1/100*	0.005
Primary Ab Reaction Time	30 min	2 h	2 h	30 min	30 min
HISTOFINE Secondary Ab and Reaction Time	30 min	30 min	30 min	30 min	30 min
AEC reaction time	25 min	2 h	2 h	30 min	60 min

	Cycle6	Cycle7	Cycle8	Cycle9	Cycle10
Primary antibody	DC-SIGN	t-bet	MHC-II	CD45	FOXP3
Clone/Product#	DC-28	SC21749(4B/10)	ERP112266	H130	236A/E7
Vendor	Santa Cruz	Santa Cruz	Abcam	BD	eBioscience
Concentration(mg/ml)	0.002	0.002	0.00026	0.005	0.0125
Primary Ab Reaction Time	30 min	30 min	30 min	30 min	30 min
HISTOFINE Secondary Ab and Reaction Time	30 min	30 min	30 min	30 min	30 min
AEC reaction time	60 min	2 h	20 min	45 min	2 h

	Cycle11	Cycle12	Cycle13	Cycle 14	Cycle 15
Primary antibody	CD4	CD8	Tbr2	Csf1R	EpCAM
Clone/Product#	4B12	C8/144b	Ab2283	SP211	E144
Vendor	Invitrogen	eBioscience	EMD	Abcam	Abcam
Concentration(mg/ml)	1/25*	0.005	0.0001	0.00064	0.000198
Primary Ab Reaction Time	2 h	30 min	30 min	30 min	60 min
HISTOFINE Secondary Ab and Reaction Time	30 min	30 min	30 min	30 min	30 min
AEC reaction time	60 min	45 min	20 min	30 min	25 min

*Dilution information provided as the concentration of the stocks is not determined by vendors

Supplementary Table S3 Lymphoid and myeloid cell types defined by multiplex IHC markers

Cell Types	IHC Markers
CD8+ T cells	CD45+CD3+CD8+
CD8+ Granzyme B+ T cells	CD45+CD3+CD8+GranzymeB+
CD8+ Ki67+ T cells	CD45+CD3+CD8+KI67+
CD8+ EOMES+ PD-1+ T cells	CD45+CD3+CD8+EOMES+PD1+
CD8+ EOMES+ PD-1- T cells	CD45+CD3+CD8+EOMES+PD1-
CD8+ EOMES- PD-1+ T cells	CD45+CD3+CD8+EOMES-PD1+
CD8+ EOMES- PD-1- T cells	CD45+CD3+CD8+EOMES-PD1-
CD4+ T cells	CD45+CD3+CD4+
CD4+ PD-1+ T cells	CD45+CD3+CD4+PD-1+
Regulatory T cells	CD45+CD3+CD4+FOXP3+
Th1	CD45+CD3+CD4+FOXP3-Tbet+RORgT-
Th2	CD45+CD3+CD4+FOXP3-GATA3+RORgT-
Th17	CD45+CD3+CD4+FOXP3-RORgT+FOXP3-
Natural killer (NK) cells	CD45+CD3-CD56+
B cells	CD45+CD3-CD56-CD20+
Tumor associated macrophage (TAM)	CD45+CD3-CD20-CD56-CD66b-Tryptase-CSF1R+
PD-L1+ CSF1-R+ cells	CD45+CD3-CD20-CD56-CD66b-Tryptase-CSF1R+PD-L1+
M1 TAM	CD45+CD3-CD20-CD56-CD66b-Tryptase-CSF1R+CD68+CD163-
PD-L1+ M1 TAM	CD45+CD3-CD20-CD56-CD66b-Tryptase-CSF1R+CD68+CD163-PD-L1+
M2 TAM	CD45+CD3-CD20-CD56-CD66b-Tryptase-CSF1R+CD68+CD163+
PD-L1+ M2 TAM	CD45+CD3-CD20-CD56-CD66b-Tryptase-CSF1R+CD68+CD163+PD-L1+
DC-SIGN+ dendritic cells	CD45+CD3-CD20-CD56-CD66b-Tryptase-MHCII+DC-SIGN+DC-LAMP-
PD-L1+ DC-SIGN+ dendritic cells	CD45+CD3/CD20/CD56-CD66b-Tryptase-MHCII+DC-SIGN+DC-LAMP-PD-L1+
DC-LAMP+ dendritic cells	CD45+CD3/CD20/CD56-CD66b-Tryptase-MHCII+DC-LAMP+
PD-L1+ DC-LAMP+ dendritic cells	CD45+CD3/CD20/CD56-CD66b-Tryptase-MHCII+DC-LAMP+PD-L1+
CD66b+ Granulocytes(Gr)	CD45+CD3/CD20/CD56-CD66b+
PD-L1+ Gr	CD45+CD3/CD20/CD56-CD66b+PD-L1+
Mast cells	CD45+CD3/CD20/CD56-CD66b-Tryptase+
PD-L1+ Mast cells	CD45+CD3/CD20/CD56-CD66b-Tryptase+PD-L1+

Supplementary Tables S4-S33

Table S4 Univariate and multivariate analysis of correlation between the density of B cells and OS < 3 yr in DCC

	Univariate Analysis			Multivariate Analysis		
**DCC**	**Odds Ratio**	**95% CI**	**p**	**Odds Ratio**	**95% CI**	**p**
**B cells:** High vs. Low	0.276	0.062, 1.233	0.092	0.251	0.054 1.177	0.08
**N1 vs. N0**	1.339	0.279, 6.434	0.715	0.969	0.18 5.2	0.97
**Margin:** Positive vs. Negative	1.212	0.212, 6.935	0.829	0.856	0.129, 5.669	0.872

Table S5 Univariate and multivariate analysis of correlation between the density of B cells and OS < 3 yr in HC

	Univariate Analysis			Multivariate Analysis		
**HC**	**Odds Ratio**	**95% CI**	**p**	**Odds Ratio**	**95% CI**	**p**
**B cells:** High vs. Low	1.714	0.219, 13.406	0.608	1.581	0.145, 17.227	0.707
**N1 vs. N0**	1.455	0.123, 17.233	0.766	0.906	0.046, 17.766	0.948
**Margin:** Positive vs. Negative	2.25	0.285, 17.759	0.442	1.613	0.152, 17.121	0.692
**Perineural Invasion** Yes vs. No	8	0.531, 20.649	0.133	5.965	0.291, 122.121	0.246
**Perivascular Invasion** Yes vs. No	2	0.174, 22.949	0.578	1.605	0.086, 29.784	0.751

Table S6 Univariate and multivariate analysis of correlation between the density of B cells and OS < 3 yr in ICC

	Univariate Analysis			Multivariate Analysis		
**ICC**	**Odds Ratio**	**95% CI**	**p**	**Odds Ratio**	**95% CI**	**p**
**B cells:** High vs. Low	0.135	0.032, 0.562	0.006	0.012	0.001, 0.25	0.004
**Tumor grade:** Moderate vs. Poor	2.758	0.572, 13.293	0.206	26.955	1.491, 487.445	0.026
**Tumor grade:** Well vs. Poor	0.583	0.044, 7.661	0.682	0.204	0.001, 34.224	0.543
**pT2 vs. T1**	2.722	0.616, 12.039	0.187	0.893	0.064, 12.477	0.933
**pT3 vs. T1**	0.519	0.046, 5.791	0.594	0.05	0, 30.044	0.359
**N1 vs. N0**	1.667	0.367, 7.566	0.508	10.266	0.18, 585.86	0.259
**Margin:** Positive vs. Negative	3.273	0.677, 15.823	0.14	11.144	0.555, 23.764	0.115

Table S7 Univariate and multivariate analysis of correlation between the density of CD4+ T cells and OS < 3 yr in DCC

	Univariate Analysis			Multivariate Analysis		
DCC	Odds Ratio	95% CI	p	Odds Ratio	95% CI	p
CD4+ T cells: High vs. Low	17.5	1.989, 153.938	0.01	19.003	2.078, 173.825	0.009
N1 vs. N0	1.339	0.279, 6.434	0.715	1.282	0.19, 8.648	0.799
Margin: Positive vs. Negative	1.212	0.212, 6.935	0.829	1.958	0.266, 14.4	0.509

Table S8 Univariate and multivariate analysis of correlation between the density of CD4+ T cells and OS < 3 yr in HC

	Univariate Analysis			Multivariate Analysis		
HC	Odds Ratio	95% CI	p	Odds Ratio	95% CI	p
CD4+ T cells: High vs. Low	6	0.532, 67.64	0.147	4.654	0.262, 82.587	0.295
N1 vs. N0	1.455	0.123, 17.233	0.766	1.662	0.083, 33.15	0.739
Margin: Positive vs. Negative	2.25	0.285, 17.759	0.442	1.406	0.134, 14.794	0.776
Perineural Invasion Yes vs. No	8	0.53, 120.649	0.133	2.975	0.111, 80.096	0.516
Perivascular Invasion Yes vs. No	2	0.174, 22.949	0.578			

Table S9 Univariate and multivariate analysis of correlation between the density of CD4+ T cells and OS < 3 yr in ICC

	Univariate Analysis			Multivariate Analysis		
ICC	Odds Ratio	95% CI	p	Odds Ratio	95% CI	p
CD4+ T cells: High vs. Low	1.857	0.522, 6.612	0.339	1.664	0.314, 8.826	0.55
Tumor grade: Moderate vs. Poor	2.758	0.572, 13.293	0.206	3.253	0.436, 24.291	0.25
Tumor grade: Well vs. Poor	0.583	0.044, 7.661	0.682	0.237	0.008, 7.026	0.405
pT2 vs. T1	2.722	0.616, 12.039	0.187	1.052	0.146, 7.583	0.96
pT3 vs. T1	0.519	0.046, 5.791	0.594	0.096	0.002, 5.093	0.248
N1 vs. N0	1.667	0.367, 7.566	0.508	4.112	0.219, 77.39	0.345
Margin: Positive vs. Negative	3.273	0.677, 15.823	0.14	2.678	0.347, 20.692	0.345

Table S10 Univariate and multivariate analysis of correlation between the density of CD8+ T cells and OS < 3 yr in DCC

DCC	Univariate Analysis			Multivariate Analysis		
	Odds Ratio	95% CI	p	Odds Ratio	95% CI	p
CD8+ T cells: High vs. Low	0.276	0.062,1.233	0.092	0.238	0.049,1.158	0.075
N1 vs. N0	1.339	0.279,6.434	0.715	1.141	0.218,5.988	0.876
Margin: Positive vs. Negative	1.212	0.212,6.935	0.829	0.65	0.093,4.53	0.663

Table S11 Univariate and multivariate analysis of correlation between the density of CD8+ T cells and OS < 3 yr in HC

HC	Univariate Analysis			Multivariate Analysis		
	Odds Ratio	95% CI	p	Odds Ratio	95% CI	P
CD8+ T cells: High vs. Low	0.167	0.532,67.64	0.147	0.181	0.015,2.24	0.183
N1 vs. N0	1.455	0.123,17.233	0.766	0.843	0.048,14.818	0.907
Margin: Positive vs. Negative	2.25	0.285,17.759	0.442	1.616	0.161,16.271	0.684
Perineural Invasion Yes vs. No	8	0.53,120.649	0.133			
Perivascular Invasion Yes vs. No	2	0.174,22.949	0.578	2.415	0.15,38.894	0.534

Table S12 Univariate and multivariate analysis of correlation between the density of CD8+ T cells and OS < 3 yr in ICC

ICC	Univariate Analysis			Multivariate Analysis		
	Odds Ratio	95% CI	p	Odds Ratio	95% CI	p
CD8+ T cells: High vs. Low	0.222	0.058, 0.858	0.029	0.218	0.038, 1.247	0.087
Tumor grade: Moderate vs. Poor	2.758	0.572,13.293	0.206	5.665	0.749,42.864	0.093
Tumor grade: Well vs. Poor	0.583	0.044,7.661	0.682	0.31	0.009,11.178	0.522
pT2 vs. T1	2.722	0.616,12.039	0.187	0.73	0.095,5.586	0.762
pT3 vs. T1	0.519	0.046,5.791	0.594	0.105	0.002,4.949	0.252
N1 vs. N0	1.667	0.367,7.566	0.508	3.969	0.238,66.318	0.337
Margin: Positive vs. Negative	3.273	0.677,15.823	0.14	2.783	0.321,24.103	0.353

Table S13 Univariate and multivariate analysis of correlation between the density of CD8+ Granzyme B+ T cells and OS < 3 yr in DCC

	Univariate Analysis			Multivariate Analysis		
DCC	Odds Ratio	95% CI	p	Odds Ratio	95% CI	p
CD8+ Granzyme B+ T cells: High vs. Low	0.144	0.027, 0.778	0.024	0.114	0.018, 0.705	0.02
N1 vs. N0	1.339	0.279 ,6.434	0.715	1.049	0.185, 5.94	0.957
Margin: Positive vs. Negative	1.212	0.212,6.935	0.829	0.494	0.06, 4.051	0.511

Table S14 Univariate and multivariate analysis of correlation between the density of CD8+ Granzyme B + T cells and OS < 3 yr in HC

	Univariate Analysis			Multivariate Analysis		
HC	Odds Ratio	95% CI	p	Odds Ratio	95% CI	P
CD8+ Granzyme B+ T cells: High vs. Low	0.583	0.075, 4.562	0.608	0.287	0.017, 4.835	0.386
N1 vs. N0	1.455	0.123,1 7.233	0.766	0.596	0.022, 15.854	0.757
Margin: Positive vs. Negative	2.25	0.285, 17.759	0.442	1.97	0.142, 27.319	0.613
Perineural Invasion Yes vs. No	8	0.53, 120.649	0.133	11.721	0.408, 336.42	0.151
Perivascular Invasion Yes vs. No	2	0.174,22.949	0.578	1.203	0.056,26.038	0.906

Table S15 Univariate and multivariate analysis of correlation between the density of CD8+ Granzyme B + T cells and OS < 3 yr in ICC

	Univariate Analysis			Multivariate Analysis		
ICC	Odds Ratio	95% CI	p	Odds Ratio	95% CI	P
CD8+ Granzyme B+ T cells: High vs. low	0.222	0.058,0.858	0.029	0.269	0.05,1.435	0.124
Tumor grade: Moderate vs. Poor	2.758	0.572,13.293	0.206	5.377	0.693,41.733	0.108
Well vs. Poor	0.583	0.044,7.661	0.682	0.381	0.011,13.593	0.597
pT2 vs. T1	2.722	0.616,12.039	0.187	0.72	0.083,6.253	0.766
pT3 vs. T1	0.519	0.046,5.791	0.594	0.041	0, 3.94	0.171
N1 vs. N0	1.667	0.367,7.566	0.508	7.283	0.285,186.205	0.23
Margin: Positive vs. Negative	3.273	0.677,15.823	0.14	2.268	0.272,18.896	0.449

Table S16 Univariate and multivariate analysis of correlation between the density of CD8+EOMES-PD-1- T cells and OS < 3 yr in DCC

DCC	Univariate Analysis			Multivariate Analysis		
	Odds Ratio	95% CI	p	Odds Ratio	95% CI	p
**CD8+EOMES-PD-1- T cells:** High vs. Low	0.476	0.117,1.944	0.301	0.501	0.122,2.062	0.338
**N1 vs. N0**	1.339	0.279,6.434	0.715	1.363	0.276,6.736	0.704
**Margin:** Positive vs. Negative	1.212	0.212,6.935	0.829	0.994	0.163,6.052	0.995

Table S17 Univariate and multivariate analysis of correlation between the density of CD8+EOMES-PD-1- T cells and OS < 3 yr in HC

HC	Univariate Analysis			Multivariate Analysis		
	Odds Ratio	95% CI	p	Odds Ratio	95 CI	P
**CD8+ EOMES-PD-1- T cells:** High vs. Low	0	0, Infinite	0.995	0	0,Inf	0.996
**N1 vs. N0**	1.455	0.123,17.233	0.766	0	0,Inf	0.998
**Margin:** Positive vs. Negative	2.25	0.285,17.759	0.442	0.751	0.02,28.261	0.877
**Perineural Invasion** Yes vs. No	8	0.53,120.649	0.133	1207613 695.628	0, Infinite	0.998
**Perivascular Invasion** Yes vs. No	2	0.174,22.949	0.578	0.751	0.02,28.261	0.877

Table S18 Univariate and multivariate analysis of correlation between the density of CD8+EOMES+PD-1+ T cells and OS < 3 yr in ICC

ICC	Univariate Analysis			Multivariate Analysis		
	Odds Ratio	95% CI	p	Odds Ratio	95% CI	P
**CD8+ EOMES-PD-1- T cells:** High vs. Low	0.076	0.016,0.358	0.001	0.049	0.006, 0.433	0.007
**N1 vs. N0**	2.758	0.572,13.293	0.206	7.673	0.791,74.398	0.079
**Margin:** Positive vs. Negative	0.583	0.044,7.661	0.682	0.32	0.004,26.69	0.614
**Perineural Invasion** Yes vs. No	2.722	0.616,12.039	0.187	0.343	0.029,4.123	0.399
**Perivascular Invasion** Yes vs. No	0.519	0.046,5.791	0.594	0.03	0,6.23	0.197
**N1**	1.667	0.367,7.566	0.508	20.544	0.421,1002.329	0.128
**Margin: Positive vs. Negative**	3.273	0.677,15.823	0.14	3.013	0.253,35.906	0.383

Table S19 Univariate and multivariate analysis of correlation between the density of PD-L1+ CSF-1R+ TAM and OS < 3 yr in DCC

DCC	Univariate Analysis			Multivariate Analysis		
	Odds Ratio	95% CI	p	Odds Ratio	95% CI	p
**PD-L1+ CSF-1R+ TAM:** High vs. Low	6.923	1.285,37.287	0.024	8.173	1.402,47.635	0.019
**N1 vs. N0**	1.339	0.279,6.434	0.715	1.323	0.235,7.463	0.751
**Margin:** Positive vs. Negative	1.212	0.212,6.935	0.829	2.208	0.326,14.96	0.417

Table S20 Univariate and multivariate analysis of correlation between the density of PD-L1+ CSF-1R+ TAM and OS < 3 yr in HC

HC	Univariate Analysis			Multivariate Analysis		
	Odds Ratio	95% CI	p	Odds Ratio	95% CI	P
**PD-L1+ CSF-1R+ TAM:** High vs. Low	1.714	0.219,13.406	0.608	5.583	0.261,119.634	0.271
**N1 vs. N0**	1.455	0.123,17.233	0.766	0.705	0.03,16.394	0.828
**Margin:** Positive vs. Negative	2.25	0.285,17.759	0.442	3.21	0.173,59.688	0.434
**Perineural Invasion** Yes vs. No	8	0.53,120.649	0.133	8.501	0.378,191.157	0.178
**Perivascular Invasion** Yes vs. No	2	0.174,22.949	0.578	2.848	0.101,80.21	0.539

Table S21 Univariate and multivariate analysis of correlation between the density of PD-L1+ CSF-1R+ TAM and OS < 3 yr in ICC

ICC	Univariate Analysis			Multivariate Analysis		
	Odds Ratio	95% CI	p	Odds Ratio	95% CI	P
**PD-L1+ CSF-1R+ TAM:** High vs. Low	7.429	1.778,31.04	0.006	10.152	1.658,62.177	0.012
**Tumor grade:** Moderate vs. Poor	2.758	0.572,13.293	0.206	2.818	0.34,23.374	0.337
**Well vs. Poor**	0.583	0.044,7.661	0.682	0.142	0.003,6.886	0.324
**pT2 vs. T1**	2.722	0.616,12.039	0.187	1.22	0.123,12.055	0.865
**pT3 vs. T1**	0.519	0.046,5.791	0.594	0.081	0.001,7.667	0.279
**N1 vs. N0**	1.667	0.367,7.566	0.508	3.09	0.111,85.791	0.506
**Margin:** Positive vs. Negative	3.273	0.677,15.823	0.14	2.568	0.248,26.578	0.429

Table S22 Univariate and multivariate analysis of correlation between the density of M1 TAM and OS<3 yr in DCC

	Univariate Analysis			Multivariate Analysis		
DCC	Odds Ratio	95% CI	p	Odds Ratio	95% CI	p
**M1 TAM:** High vs. Low	0.476	0.117,1.944	0.301	0.449	0.105,1.927	0.281
**N1 vs. N0**	1.339	0.279,6.434	0.715	1.095	0.213,5.628	0.913
**Margin:** Positive vs. Negative	1.212	0.212,6.935	0.829	0.917	0.149,5.646	0.926

Table S23 Univariate and multivariate analysis of correlation between the density of M1 TAM and OS < 3 yr in HC

	Univariate Analysis			Multivariate Analysis		
HC	Odds Ratio	95% CI	p	Odds Ratio	95% CI	P
**M1 TAM:** High vs. Low	0.583	0.075,4.562	0.608	0.527	0.062,4.449	0.556
**N1 vs. N0**	1.455	0.123,17.233	0.766	1.015	0.063,16.363	0.992
**Margin:** Positive vs. Negative	2.25	0.285,17.759	0.442	2.538	0.285,22.592	0.404
**Perineural Invasion** Yes vs. No	8	0.53,120.649	0.133			
**Perivascular Invasion** Yes vs. No	2	0.174,22.949	0.578	2.14	0.142,32.208	0.582

Table S24 Univariate and multivariate analysis of correlation between the density of M1 TAM and OS < 3 yr in ICC

	Univariate Analysis			Multivariate Analysis		
ICC	Odds Ratio	95% CI	p	Odds Ratio	95% CI	P
**M1 TAM:** High vs. Low	0.222	0.058,0.858	0.029	0.208	0.039,1.104	0.065
**Tumor grade: Moderate vs. Poor**	2.758	0.572,13.293	0.206	5.432	0.66,44.682	0.115
**Well vs. Poor**	0.583	0.044,7.661	0.682	0.304	0.01,8.9	0.49
**pT2 vs. T1**	2.722	0.616,12.039	0.187	0.793	0.089,7.036	0.835
**pT3 vs. T1**	0.519	0.046,5.791	0.594	0.044	0,4.674	0.189
**N1 vs. N0**	1.667	0.367,7.566	0.508	5.724	0.206,158.813	0.303
**Margin:** Positive vs. Negative	3.273	0.677,15.823	0.14	2.482	0.295,20.894	0.403

Table S25 Univariate and multivariate analysis of correlation between the density of PD-L1+ M1 TAM and OS < 3 yr in DCC

DCC	Univariate Analysis			Multivariate Analysis		
	Odds Ratio	95% CI	p	Odds Ratio	95% CI	p
PD-L1+ M1 TAM: High vs. Low	6.923	1.285,37.287	0.024	7.07	1.303,38.349	0.023
N1 vs. N0	1.339	0.279,6.434	0.365			
Margin: Positive vs. Negative	1.212	0.212,6.935	0.829	1.423	0.221,9.15	0.71

Table S26 Univariate and multivariate analysis of correlation between the density of PD-L1+ M1 TAM and OS < 3 yr in HC

HC	Univariate Analysis			Multivariate Analysis		
	Odds Ratio	95% CI	p	Odds Ratio	95% CI	P
PD-L1+ M1 TAM: High vs. Low	1.714	0.219,13.406	0.608	0.665	0.041,10.784	0.774
N1 vs. N0	1.455	0.123,17.233	0.766	0.738	0.037,14.815	0.843
Margin: Positive vs. Negative	2.25	0.285,17.759	0.442	1.545	0.15,15.946	0.715
Perineural Invasion Yes vs. No	8	0.53,120.649	0.133	9.074	0.247,333.173	0.23
Perivascular Invasion Yes vs. No	2	0.174,22.949	0.578	1.543	0.086,27.63	0.768

Table S27 Univariate and multivariate analysis of correlation between the density of PD-L1+ M1 TAM and OS < 3 yr in ICC

ICC	Univariate Analysis			Multivariate Analysis		
	Odds Ratio	95% CI	p	Odds Ratio	95% CI	P
PD-L1+ M1 TAM: High vs. Low	2.852	0.777,10.467	0.114	6.166	0.875,43.46	0.068
Tumor grade: Moderate vs. Poor	2.758	0.572,13.293	0.206	3.143	0.432,22.868	0.258
Well vs. Poor	0.583	0.044,7.661	0.682	0.162	0.003,8.648	0.369
pT2 vs. T1	2.722	0.616,12.039	0.187	1.86	0.213,16.217	0.574
pT3 vs. T1	0.519	0.046,5.791	0.594	0.086	0.002,4.201	0.216
N1 vs. N0	1.667	0.367,7.566	0.508	9.247	0.523,163.547	0.129
Margin: Positive vs. Negative	3.273	0.677,15.823	0.14	2.321	0.288,18.706	0.429

Table S28 Univariate and multivariate analysis of correlation between the density of M2 TAM and OS < 3 yr in DCC

DCC	Univariate Analysis			Multivariate Analysis		
	Odds Ratio	95% CI	p	Odds Ratio	95% CI	p
M2 TAM: High vs. Low	6.923	1.285,37.287	0.024	7.473	1.365,40.907	0.02
N1 vs. N0	1.339	0.279,6.434	0.715	1.045	0.19,5.745	0.96
Margin: Positive vs. Negative	1.212	0.212,6.935	0.829	1.072	0.157,7.329	0.943

Table S29 Univariate and multivariate analysis of correlation between the density of M2 TAM cells and OS < 3 yr in HC

HC	Univariate Analysis			Multivariate Analysis		
	Odds Ratio	95% CI	p	Odds Ratio	95 CI	P
M2 TAM: High vs. Low	6	0.532,67.64	0.147	5.194	0.213,126.625	0.312
N1 vs. N0	1.455	0.123,17.233	0.766	1.457	0.05,42.864	0.827
Margin: Positive vs. Negative	2.25	0.285,17.759	0.442	0.877	0.055,13.911	0.926
Perineural Invasion Yes vs. No	8	0.53,120.649	0.133	2.563	0.083,79.058	0.591
Perivascular Invasion Yes vs. No	2	0.174,22.949	0.578	1.753	0.092,33.309	0.709

Table S30 Univariate and multivariate analysis of correlation between the density of M2 TAM and OS < 3 yr in ICC

ICC	Univariate Analysis			Multivariate Analysis		
	Odds Ratio	95% CI	p	Odds Ratio	95% CI	P
M2 TAM: High vs. Low	1.227	0.35,4.307	0.749	2.35	0.467,11.831	0.3
Tumor grade: Moderate vs. Poor	2.758	0.572,13.293	0.206	4.151	0.603,28.584	0.148
Well vs. Poor	0.583	0.044,7.661	0.682	0.192	0.006,6.056	0.349
pT2 vs. T1	2.722	0.616,12.039	0.187	1.479	0.214,10.216	0.691
pT3 vs. T1	0.519	0.046,5.791	0.594	0.078	0.001,4.671	0.222
N1 vs. N0	1.771	0.392,8.002	0.458	6.411	0.331,124.16	0.219
Margin: Positive vs. Negative	3.273	0.677,15.823	0.14	1.455	0.238,8.91	0.685

Table S31 Univariate and multivariate analysis of correlation between the density of PD-L1+ M2 TAM and OS < 3 yr in DCC

	Univariate Analysis			Multivariate Analysis		
**DCC**	**Odds Ratio**	**95% CI**	**p**	**Odds Ratio**	**95% CI**	**p**
**PD-L1+ M2 TAM:** High vs. Low	314366015.311	0,Inf	0.993	314366015.306	0,Inf	0.993
**N1 vs. N0**	1.339	0.279,6.434	0.715	1	0.153,6.534	1
**Margin:** Positive vs. Negative	1.212	0.212,6.935	0.829	1	0.114,8.736	1

Table S32 Univariate and multivariate analysis of correlation between the density of PD-L1+ M2 TAM and OS < 3 yr in HC

	Univariate Analysis			Multivariate Analysis		
**HC**	**Odds Ratio**	**95% CI**	**p**	**Odds Ratio**	**95% CI**	**P**
**PD-L1+ M2 TAM:** High vs. Low	0.583	0.075,4.562	0.608	0.244	0.012,5.12	0.364
**N1 vs. N0**	1.455	0.123,17.233	0.766	0.371	0.011,12.772	0.583
**Margin:** Positive vs. Negative	2.25	0.285,17.759	0.442	1.3	0.116,14.542	0.831
**Perineural Invasion** Yes vs. No	8	0.53,120.649	0.133	13.39	0.366,489.914	0.158
**Perivascular Invasion** Yes vs. No	2	0.174,22.949	0.578	2.002	0.088,45.646	0.664

Table S33 Univariate and multivariate analysis of correlation between the density of PD-L1+ M2 TAM cells and OS < 3 yr in ICC

	Univariate Analysis			Multivariate Analysis		
**ICC**	**Odds Ratio**	**95% CI**	**p**	**Odds Ratio**	**95% CI**	**P**
**PD-L1+ M2 TAM:** High vs. Low	7.429	1.778,31.04	0.006	10.812	1.63,71.735	0.014
**Tumor grade:** Moderate vs. Poor	2.758	0.572,13.293	0.206	2.694	0.318,22.862	0.364
**Well vs. Poor**	0.583	0.044,7.661	0.682	0.167	0.002,12.118	0.413
**pT2 vs. T1**	2.722	0.616,12.039	0.187	0.884	0.101,7.741	0.912
**pT3 vs. T1**	0.519	0.046,5.791	0.594	0.057	0.001,6.325	0.233
**N1 vs. N0**	1.667	0.367,7.566	0.508	13.68	0.419,447.119	0.141
**Margin:** Positive vs. Negative	3.273	0.677,15.823	0.14	2.007	0.213,18.946	0.543
